# Liquid-Liquid Chromatography Separation of Guaiane-Type Sesquiterpene Lactones from *Ferula penninervis* Regel & Schmalh. and Evaluation of Their In Vitro Cytotoxic and Melanin Inhibitory Potential

**DOI:** 10.3390/ijms221910717

**Published:** 2021-10-03

**Authors:** Simon Vlad Luca, Katarzyna Gaweł-Bęben, Marcelina Strzępek-Gomółka, Ainur Jumabayeva, Zuriyadda Sakipova, Jianbo Xiao, Laurence Marcourt, Jean-Luc Wolfender, Krystyna Skalicka-Woźniak

**Affiliations:** 1 Biothermodynamics, TUM School of Life Sciences, Technical University of Munich, 85354 Freising, Germany; 2 Department of Pharmacognosy, “Grigore T. Popa” University of Medicine and Pharmacy Iasi, 700115 Iasi, Romania; 3 Department of Cosmetology, University of Information Technology and Management in Rzeszów, 35-225 Rzeszów, Poland; mstrzepek@wsiz.edu.pl; 4 School of Pharmacy, Kazakh National Medical University Named after S.D. Asfendiyarov (KazNMU), Almaty 050012, Kazakhstan; ainur.j0603@gmail.com (A.J.); sakipova.z@kaznmu.kz (Z.S.); 5 Nutrition and Bromatology Group, Department of Analytical Chemistry and Food Science, Faculty of Food Science and Technology, Ourense Campus, University of Vigo, E-32004 Ourense, Spain; jianboxiao@yahoo.com; 6 School of Pharmaceutical Sciences, University of Geneva, CMU-Rue Michel-Servet 1, 1211 Geneva 4, Switzerland; Laurence.Marcourt@unige.ch (L.M.); jean-luc.wolfender@unige.ch (J.-L.W.); 7 Institute of Pharmaceutical Sciences of Western Switzerland, IPSWS, University of Geneva, CMU, 1211 Geneva 4, Switzerland; 8 Independent Laboratory of Natural Products Chemistry, Medical University of Lublin, 20-093 Lublin, Poland; kskalicka@pharmacognosy.org

**Keywords:** *Ferula penninervis*, sesquiterpene lactones, ferupennin P-Q, countercurrent chromatography, prostate cancer, melanin inhibition

## Abstract

*Ferula penninervis* Regel & Schmalh. is a perennial plant used in Kazakh traditional folk medicine to treat epilepsy, neurosis, rheumatism, gastroduodenal ulcers, dyspepsia, wounds, abscesses or tumors. The aim of this work was to isolate series of sesquiterpene lactones from a crude methanolic root extract and investigate their in vitro cytotoxic potential against androgen-dependent prostate cancer LNCaP and epithelial prostate PNT2 cells, as well as to evaluate their melanin production inhibitory effects in murine melanoma B16F10 cells stimulated with α-melanocyte-stimulating hormone (αMSH). Two new (penninervin P and penninervin Q) and five known (olgin, laferin, olgoferin, oferin and daucoguainolactone F) guaiane-type sesquiterpene lactones were isolated with the use of a simple and fast liquid-liquid chromatography method. Olgin and laferin showed the most promising cytotoxic effects in LNCaP cells (IC_50_ of 31.03 and 23.26 μg/mL, respectively). Additionally, olgin, laferin, olgoferin, and oferin (10 μg/mL) potently impaired melanin release (40.67–65.48% of αMSH + cells) without influencing the viability of B16F10 cells. In summary, our findings might indicate that guaiane-type sesquiterpene lactones from *F. penninervis* could be regarded as promising candidates for further research in discovering new therapeutic agents with anti-prostate cancer and skin depigmentation properties.

## 1. Introduction

*Ferula* L. (Apiaceae) comprises around 180 species of perennial flowering plants distributed throughout the mountainous or arid regions of the Mediterranean basin of central and south-west Asia, especially the former Soviet Union, Iran, North India and Northwest China [1,2]. Several species are used as food flavoring agents or traditional local remedies for treating asthma, bronchitis, whooping cough, epilepsy, stomachache, flatulence, skin infections, rheumatoid arthritis, and ulcer [3,4,5,6]. The genus is acknowledged as a good source of biologically active constituents, including simple, sesquiterpene and dimeric coumarins, as well as guaiane-, daucane-, humulane- and himachalane-type sesquiterpenes [7,8]. *Ferula* species are also very popular for their oleoresin content, a complex mixture of different volatile and non-volatile constituents such as phenylpropanoids, other aromatic compounds and essential oils [9]. A wide range of promising pharmacological effects, namely anti-inflammatory, neuroprotective, antidiabetic, antiviral, antibacterial, antiprotozoal, anti-ulcer, hepatoprotective and cytotoxic, were documented for different *Ferula* extracts or isolated constituents [2,7,10,11,12].

*Ferula penninervis* Regel & Schmalh. (syn. *Ferula olgae* Regel & Schmalh., feather-veined giant fennel) is a perennial herbaceous plant species spread in the dry and hot regions of Central Asia [13]. In Kazakh traditional folk medicine, the roots are used internally (decocts) or externally (tinctures, compresses) to treat epilepsy, neurosis, rheumatism and various inflammatory diseases, gastroduodenal ulcers, dyspepsia, wounds, abscesses and tumours [14]. To date, numerous sesquiterpene lactones, including olgin, olgoferin, oferin, laferin, talassins A-B, ferolide, penninervin, ferupennins A-O, lasolide, isolasolide, decipienin F, and carmenin have been isolated from the roots of *F. penninervis* [12,15,16,17]. However, to the best of our knowledge, the biological potential of this species and its phytochemical constituents has been almost completely neglected.

To improve the current knowledge around *F. penninervis*, this study was initially designed to investigate the suitability of liquid-liquid chromatography (LLC) to isolate sesquiterpene lactones from a crude methanolic root extract. Next, the in vitro cytotoxic effects of the raw extract were screened by the neutral red uptake assay against cell lines derived from the gastrointestinal tract (gastric cancer N87 cells, colorectal cancer Caco-2 and HT-29 cells), prostate (prostate epithelial PNT2 cells, androgen-dependent prostate cancer LNCaP cells, androgen-insensitive prostate cancer DU145 and PC3 cells), and skin (BJ fibroblasts, HaCaT keratinocytes, squamous cell cancer SCC-15 cells, malignant melanoma A375 and B16F10 cells). The influence of the isolated sesquiterpene lactones on LNCaP and PNT2 cell viability was further evaluated. Lastly, the melanin production inhibitory effects of *F. penninervis* methanolic root extract and its constituents were assessed in B16F10 cells stimulated with α-melanocyte-stimulating hormone (αMSH).

## 2. Results and Discussion

### 2.1. Liquid-Liquid Chromatography Separation of Sesquiterpene Lactones from Ferula penninervis Methanolic Root Extract

To date, numerous sesquiterpene lactones have been isolated from the roots of *F. penninervis* [12,15,16,17]. However, all the previous separation studies have been carried out with the use of conventional preparative chromatographic procedures, such as thin layer chromatography, column chromatography or gel permeation chromatography, which are known to be solvent and time consuming. Support-free liquid-liquid chromatography (LLC), commonly referred as countercurrent chromatography (CCC) and centrifugal partition chromatography (CPC), is considered a hybrid technique between liquid-liquid extraction (due to its partitioning separation mechanisms and high loading capacity) and solid-liquid chromatography (due to its very high selectivity). The ‘column’ in LLC is mounted either on a single axis rotor (in CPC) or double axis rotor (in CCC) and it is subjected to a centrifugal field that allows the stationary liquid phase to be kept inside the column, while the mobile liquid phase, immiscible with the first one, is pumped through it [18,19,20]. Since there is no solid sorbent, no irreversible adsorption can practically occur, whereas the costs of purification are relatively low, as expensive solid stationary phases, time intensive column packing procedures or high-purity solvents are not required [21,22]. The tailor-made mannerly preparation of the biphasic solvent systems as well as the numerous operating mode possibilities (classical ascending or descending elution, gradient elution, extrusion elution, recycling elution, continuous elution, (multiple) dual mode elution) make LLC a highly versatile and adaptable separation technique [23].

**Figure 1:**
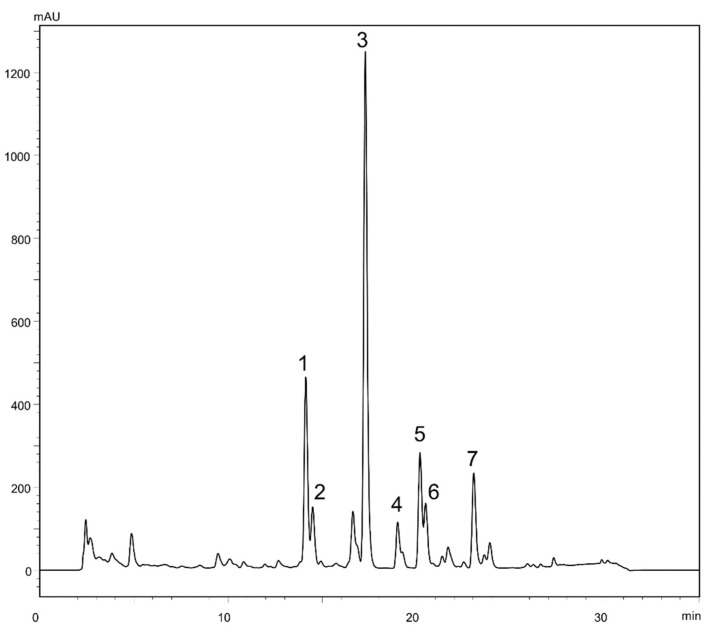
HPLC-DAD chromatogram of *Ferula penninervis* methanolic root extract. olgin (**1**), ferupennin P (**2**), laferin (**3**), olgoferin (**4**), oferin (**5**), ferupennin Q (**6**) and daucoguainolactone F (**7**).

In order to achieve a successful purification of target compounds using LLC, a careful selection of the biphasic solvent system is required. Its suitability is quickly pre-evaluated by assessing the partition coefficient (*P_i_*) values and the separation factors (*α_ij_*). *P_i_* (defined as the ratio between the concentration of the target in the stationary phase and that in the mobile phase) is considered to be optimum within the range of 0.4–2.5, whereas good separation factors (*α_ij_ = P_i_/P_j_, P_i_ > P_j_*) should be higher than 1.5 [21,24]. Various mixtures of HEMWat have been successfully used for the LLC separation of several sesquiterpene lactones, such as eupalinolides A-B from *Eupatorium lindleyanum* DC. [25] and lactucopicrin from *Cichorium glandulosum* Boiss. et Huet. [26]. Therefore, for the seven target constituents from the roots of *F. penninervis* (Figure 1) which were previously shown to be rich in sesquiterpene lactones [12], four different HEMWat systems were screened (Table 1).

**Figure 2:**
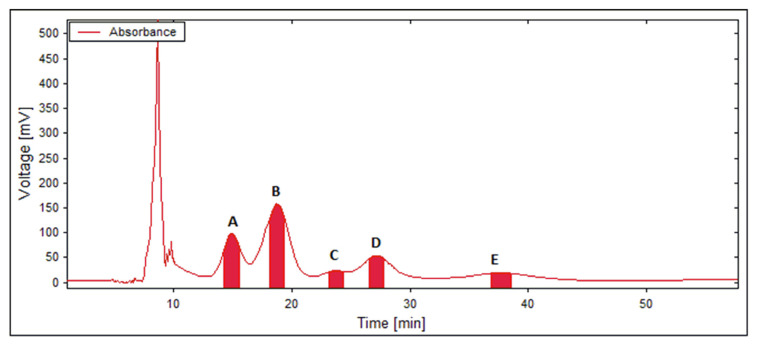
On-line chromatogram of the LLC separation of the target constituents from *Ferula penninervis* methanolic root extract. Biphasic solvent system: *n*-hexane/ethyl acetate/methanol/water (3/2/3/2, *v/v/v/v*); Elution mode: reversed-phase (head-to-tail), Unit: CCC; Column volume: 136 mL; Flow-rate: 6 mL/min; Rotation speed: 1600 rpm; Injection volume: 6 mL; Extract concentration: 40 mg/mL; UV: 254 nm. The collected fractions were labelled with letters (**A**–**E**).

Since suitable *P_i_* values (between 0.44 and 1.89) were provided only with HEMWat 3/2/3/2 (*v*/*v*/*v*/*v*), the LLC separations were performed with this solvent composition. After four repeated experiments on a CCC unit (under the experimental conditions described in Figure 2), five fractions were yielded, as follows: Fr. A (12.5 mg), Fr. B (9.7 mg), Fr. C (4.2 mg), Fr. D (8.8 mg) and Fr. E (4.9 mg). After checking the UV spectral purity of all collected fractions by HPLC-DAD, it was noticed that Fr. A contained a 5:1 mixture of compounds **1** and **2**, Fr. B contained compound **3** (95.7%), Fr. C contained compound **4** (95.4%), Fr. D contained a 5:2 mixture of compounds **5** and **6** and Fr. E contained compound **7** (98.4%). The fact that compounds **1**–**2** and **5**–**6** were not resolved under the LLC experimental conditions was expected from their separation factors α_21_ = 1.1 and α_65_ = 1.0 (Table 1). Hence, Frs. A and D were further purified by semi-preparative HPLC, under isocratic elution with 45% and 55% methanol, respectively, yielding 5.9 mg of compound **1** (98.7%), 1.0 mg of compound **2** (95.1%), 3.1 mg of compound **5** (98.0%) and 1.3 mg of compound **6** (91.1%). Thus, three of the seven target compounds were directly afforded with satisfactory purities at the end of the LLC processing steps. Additionally, for the purification of the other four targets, solid-liquid chromatography (namely preparative HPLC) was complementarily employed.

### 2.2. Structure Elucidation of the New Guaiane-Type Sesquiterpene Lactones

**Figure 3:**
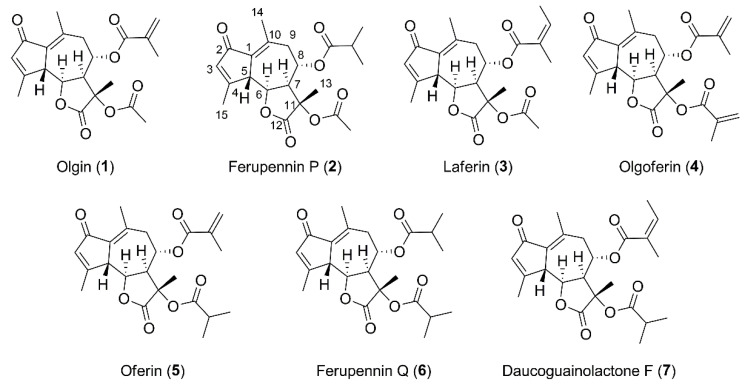
Chemical structures of the seven guaiane-type sesquiterpene lactones isolated from *Ferula penninervis* methanolic root extract.

Two new and five known guaiane-type sesquiterpene lactones (Figure 3 and Figure S1) were isolated from *F. penninervis* methanolic root extract. The known compounds were identified as olgin (**1**), laferin (**3**), olgoferin (**4**), oferin **(5**) and daucoguainolactone F (**7**) by comparing their spectral data with those reported in spectral libraries and literature [15,27,28].

Compound **2** had a protonated ion [M + H]^+^ at *m/*z 391.1773 (calcd. for C_21_H_27_O_7_^+^_,_ *m/z* 391.1751, Δ = 5.62 ppm), as detected by HRMS in positive ionization mode. The ESI-MS/MS fragment ions at *m/z* 331.1561 [M–acetyl–H_2_O + H]^+^, 261.1134 [M–acetyl–*i*butyroyl–H_2_O + H]^+^ and 243.1031 [M–acetyl–*i*butyroyl–2 × H_2_O + H]^+^ suggested the presence of one acetyl and one isobutyroyl group attached to a guaiane-type sesquiterpene lactone skeleton (Figure S2). The NMR data of compound **2** (Table 2, Figures S3–S8) showed close similarities with olgin (**1**), except that the methacrylate group present in the C-8 position of olgin was replaced by an isobutyryl group [δ_H_ 2.61 (1H, hept, *J* = 7.0 Hz, H-8b), 1.21 (3H, d, *J* = 7.0 Hz, H-8d), and 1.24 (3H, d, *J* = 7.0 Hz, H-8c)]. It was confirmed by the HMBC correlation from H-8b and H-8 at δ_H_ 5.60 (1H, td, *J* = 10.9, 3.3 Hz, H-8) to C-8a (δ_C_ 177.3). The ROESY correlations from H-5 at δ_H_ 3.94 (1H, d, *J* = 11.4 Hz, H-5) to H-8 and H-13 at δ_H_ 1.61 (3H, s, H-13) and from H-6 at δ_H_ 4.69 (1H, t, *J* = 11.4, 9.7 Hz, H-6) to H-7 at δ_H_ 3.63 (1H, dd, *J* = 10.9, 9.7 Hz, H-7) indicated that the relative configuration was the same than olgin. Based on these findings, the new compound **2** was assigned as 2-oxo-8*α*-isobutyroyloxy-11*α*-acetoxy-5*β*H,6*α*H,7*α*H-gua-1(10),3-diene-6,12-olide (ferupennin P, Figure 3).

The HRMS (positive ionization mode) spectrum of compound **6** (Figure S9) showed a molecular ion [M + H]^+^ at *m/*z 419.2066 (calcd. for C_23_H_31_O_7_^+^_,_ *m/z* 419.2064, Δ = 0.48 ppm). The ESI-MS/MS fragment ions at *m/z* 349.1636 [M–*i*butyroyl+H]^+^, 331.1534 [M–*i*butyroyl–H_2_O + H]^+^, 261.1118 [M–2×*i*butyroyl–H_2_O + H]^+^ and 243.1015 [M–2 × i-But–2 × H_2_O + H]^+^ suggested the presence of two isobutyroyl groups attached to the same guaiane-type sesquiterpene lactone skeleton as in compound **2**. The NMR data of compound **6** (Table 2, Figures S10–S14) confirmed the presence of two isobutyryl groups, the first one at δ_H_ 2.60 (1H, hept, *J* = 6.9 Hz, H-8b), 1.23 (3H, d, *J* = 6.9 Hz, H- 8c), and 1.20 (3H, d, *J* = 6.9 Hz, H-8d), and the second one at δ_H_ 2.60 (1H, hept, *J* = 6.9 Hz, H-11b), 1.20 (3H, d, *J* = 6.9 Hz, H-11c), and 1.19 (3H, d, *J* = 6.9 Hz, H-11d). The HMBC correlation from H-8 at δ_H_ 5.60 (1H, td, *J* = 11.1, 3.3 Hz, H-8) and H-8b (or H-11b) to C-8a (or C-11a) at δ_C_ (177.3) confirmed that one of the isobutyryl group was linked to C-8. The second one was linked to the remaining oxygenated carbon as indicated by the chemical shift of C-11 (δ_C_ 79.3) and the HMBC correlation from methyl at δ_H_ 1.61 (3H, s, H-13) to C-11, C-7 at δ_C_ (48.8) and C-12 at δ_C_ (175.6). The ROESY correlations were the same as those observed for the whole series of isolated compounds. The new compound **6** was assigned as 2-oxo-8*α*,11*α*-diisobutyroyloxy-5*β*H,6*α*H,7*α*H-gua-1(10),3-diene-6,12-olide (ferupennin Q, Figure 3).

### 2.3. Effects on Gastrointestinal, Prostate and Skin Cancer and Non-Cancer Cell Viability

According to the World Health Organization (WHO), cancer is the leading cause of death worldwide after cardiovascular diseases, accounting for an estimated 9.6 million deaths in 2018 [29]. Colorectal (1.80 million cases), prostate (1.28 million cases), skin (non-melanoma, 1.04 million cases) and stomach (1.03 million cases) carcinomas are among the most common types of cancer [29]. Aside from radiotherapy, surgery and immunotherapy, chemotherapy is still the main cornerstone in cancer management. Furthermore, over 60% of the currently used anti-cancer chemotherapeutics are represented by natural products, natural product botanicals, natural product derivatives or natural product mimics [30,31]. In addition, plants have a long history of use in the treatment of cancer, although many of their efficiency claims are skeptically viewed due to the overall poor definition of cancer as a disease in the traditional medicinal context [32].

Since one of the most prominent bioactivity features of the *Ferula* species is represented by their cytotoxicity against cancer cells [33,34,35,36,37], we initially assessed the in vitro cytotoxicity of *F. penninervis* methanolic root extract against cancer and non-cancer cell lines. The cell lines used in this study were derived from the gastrointestinal tract (gastric cancer N87 cells, colorectal cancer Caco-2 and HT-29 cells), prostate (prostate epithelial PNT2 cells, androgen-dependent prostate cancer LNCaP cells, androgen-insensitive prostate cancer DU145 and PC3 cells), and skin (BJ fibroblasts, HaCaT keratinocytes, squamous cell cancer SCC-15 cells, malignant melanoma A375 and B16F10 cells). This panel of cell lines is commonly used to investigate new agents with potential gastrointestinal, prostate and skin anti-cancer activity.

#### 2.3.1. Cytotoxic Activity of *Ferula penninervis* Methanolic Root Extract

**Figure 4:**
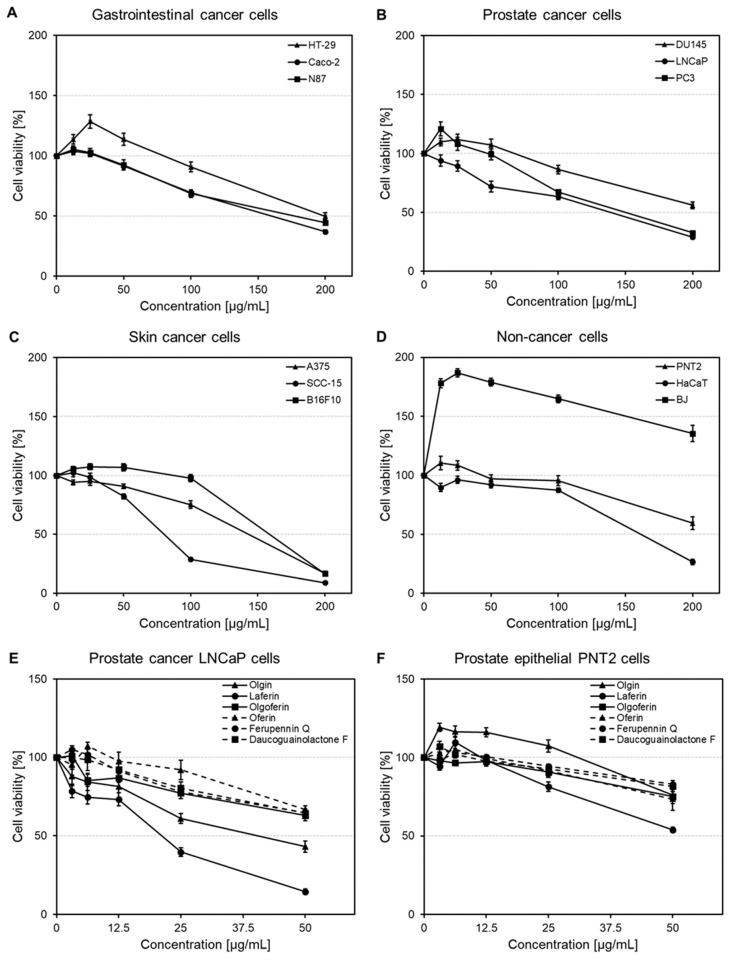
Concentration-response curves showing the effect of *Ferula penninervis* methanolic root extract against gastrointestinal cancer cells (**A**), prostate cancer cells (**B**), skin cancer cells (**C**) and non-cancer cells (**D**) and the effect of isolated sesquiterpene lactones against prostate cancer LNCaP cells (**E**) and prostate epithelial PNT2 cells (**F**). Cells were seeded in 96-well plates (3 × 10^3^/well) and treated with increasing concentrations of extract (12.5–200 μg/mL) or sesquiterpene lactones (3.125–50 μg/mL); after 48 h, the cell viability was evaluated using neutral red uptake assay. Data are expressed as percentage change in viability in comparison to the vehicle (DMSO) treated control group. Each point represents the mean ± S.E.M. of three independent experiments performed in triplicates.

The viability of gastrointestinal cancer cells was concentration-dependently reduced over the range of 12.5–200 μg/mL (Figure 4A). At the highest tested concentration, the percentages of viable cells were decreased to 44.37 ± 1.52%, 36.91 ± 1.50% and 49.76 ± 2.83% in N87, Caco-2 and HT-29 cells, respectively. According to the calculated IC_50_ values, Caco-2 were the most sensitive cells (Table 3). The survival rates of prostate cancer cells also declined in a concentration-dependent manner (Figure 4B and Figure S15). At 200 μg/mL, *F. penninervis* methanolic root extract reduced DU145, LNCaP and PC3 cell viabilities to 56.02 ± 2.88%, 29.08 ± 1.63% and 32.64 ± 1.47%, respectively. However, reductions of the cell viability at lower concentrations were observed only for LNCaP cell lines (71.92 ± 4.64% cell viability at 50 μg/mL). Nevertheless, based on the IC_50_ values, LNCaP and PC3 were the most sensitive prostate cancer cells, with comparable IC_50_ values (Table 3).

The viability of non-cancer epithelial prostate PNT2 cells was not affected up to 100 μg/mL (viabilities >95%). However, at 200 μg/mL, a significant reduction of PNT2 cell viability to 59.46 ± 5.22% was observed (Figure 4D). The effects produced by the crude extract against skin cancer cells were more drastic, especially at higher concentrations (Figure 4C). At 200 μg/mL, A375, SCC-15 and B16F10 cell viabilities were decreased to 16.74 ± 1.70%, 8.96 ± 0.70% and 16.59 ± 2.14%, respectively. According to the IC_50_ values, the following decreasing order of activity was observed: SCC-15 > B16F10 > A375 cells (Table 3).

On the other hand, skin-derived non-cancer cells were influenced in different manners. Up to 100 μg/mL, the viability of HaCaT keratinocytes was not reduced to less than 87%; however, a concentration of 200 μg/mL reduced their viability to 26.51 ± 2.37% (Figure 4D); this might suggest a potential skin irritating effect at high doses. Nevertheless, the treatment of BJ fibroblasts with *F. penninervis* methanolic root extract (12.5–200 μg/mL) significantly increased the number of viable fibroblasts as compared to the vehicle control treated cells (Figure 4D); the fibroblast growth-stimulating activity of plant extracts could suggest their potential regenerative and anti-aging properties [38,39,40]. Overall, our data are in agreement with previous reports that assessed the cytotoxic effects of other *Ferula* species (e.g., *F. gummosa* Boiss., *F. szowitsiana* DC., *F. persica* Willd., *F. hezarlalezarica* Ajani, *F. hirtella* Boiss., *F. oopoda* Boiss) against different panels of cancer cells (e.g., breast cancer MCF-7, squamous cancer BHY, malignant melanoma SKMEL-3, liver cancer HepG2, lung cancer A549, colon cancer HT-29) [33,34,35,36,37]. For instance, the methanolic (80%) extracts of *F. szowitsiana* and *F. hirtella* exhibited IC_50_ values ranging from 36 μg/mL and 235 μg/mL against HT-29, MCF-7, A549 and HepG2 cells [37].

#### 2.3.2. Cytotoxic Activity of Guaiane-Type Sesquiterpene Lactones

Despite the fact that the intrinsic cytotoxicity of the sesquiterpene lactone nucleus is already established [41], the effects of *Ferula* sesquiterpenes against cancer cells were scarcely reported in previous investigations [42,43,44]. Therefore, olgin, laferin, olgoferin, oferin, ferupennin Q and daucoguaianolactone F were next assessed for their cytotoxicity against LNCaP cells in comparison to prostate epithelial PNT2 control cells. Among all the screened cell lines, the ones derived from the prostate were selected for this step, since the crude methanolic root extract of *F. penninervis* showed better selectivity in reducing the survival rates of the cancer cells (especially LNCaP cells) as compared to the non-cancer cells.

At 50 µg/mL, olgin and laferin reduced LNCaP cell viability to 43.10 ± 3.52% and 14.40 ± 1.69%, respectively, while the other tested compounds did not decrease the number of viable LNCaP cells to less than 63% at the same concentration (Figure 4E and Figure S16). The treatment with the positive control (5-FU, 5 μg/mL) showed a reduction of LNCaP cell viability to 50.65 ± 3.28%. The IC_50_ values were extracted only for olgin (31.03 ± 1.12 μg/mL) and laferin (23.26 ± 0.81 μg/mL), while the IC_50_ values of the remaining tested compounds were estimated as > 50 μg/mL. Moreover, olgin did not significantly affect the viability of non-cancer prostate epithelial PNT2 control cells (Figure 4F). In contrast, laferin reduced PNT2 cell survival to 53.85 ± 1.71% at the highest tested concentration (50 µg/mL), which might indicate a putative toxicity at high doses in non-cancer cells.

Globally, our results are in agreement with the very few reports that previously evaluated the cytotoxicity of *Ferula* sesquiterpenes. For instance, ferutinin isolated from *F. tenuissima* Hub.-Mor & Peşmen. exhibited cytotoxic activity against PC3 cells, with an IC_50_ value of 19.7 μM [42]. In another study, the guaiane-type lactone dehydrooopodin isolated from *F. oopoda* (Boiss. & Buhse) Boiss. revealed significant cytotoxicity, with IC_50_ values of 5 and 15 µM against erythroleukemia K562 and MCF-7 cells, respectively [43]. A eudesmane-type sesquiterperne lactone isolated for the first time by Suzuki et al. [44] from *F. varia* (Schrenk) Trautv. showed a 4.6-fold more potent cytotoxicity against the multidrug-resistant nasopharynx epidermoid cancer KB-C2 cells (IC_50_ = 15.7 μg/mL) than against the drug sensitive cancer KB cells (IC_50_ = 72.8 μg/mL) [44].

### 2.4. Influence on Melanin Synthesis and Release in αMSH-Stimulated Melanoma B16F10 Cells

Produced in the melanocytes and then released and transferred to keratinocytes, melanin is the main pigment of skin. However, abnormal production and accumulation of melanin due to excessive sun exposure can produce hyperpigmentation skin disorders such as melasma, senile lentigo or Riehl melanosis [45,46]. Disruption of melanogenesis by depigmentation (skin-lightening) agents has been targeted for therapies or protection from hyperpigmentation disorders. Nevertheless, the currently available drugs, such as arbutin, hydroquinone or kojic acid, are banned in numerous countries, since they have been reported to cause permanent depigmentation, skin cancer or dermatitis [46,47]. Therefore, there is a real need for discovering novel, safe and effective depigmentation agents.

**Figure 5:**
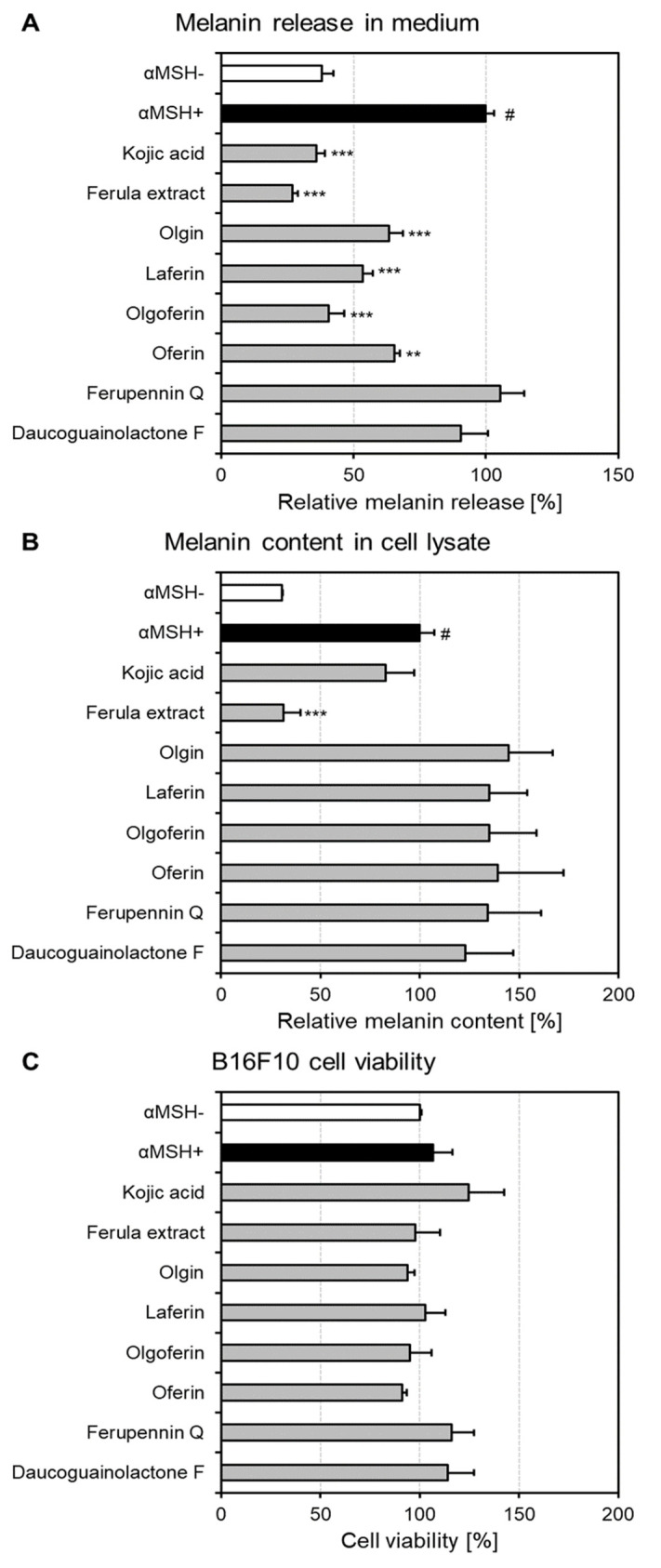
Influence of *Ferula penninervis* root extract and isolated sesquiterpene lactones on melanin release in conditioned medium (**A**), melanin content in cell lysate (**B**) and cell viability (**C**) in α-MSH-stimulated murine melanoma B16F10 cells. B16F10 cells were seeded in 6-well plates (0.5 × 10^5^/well) and treated with extract (100 μg/mL) or sesquiterpene lactones (10 μg/mL); melanin production was stimulated with α-MSH (10 nM). Kojic acid (100 μg/mL) was used as positive control. After 72 h, the melanin release/content and cell viability were assessed. Data are expressed as the percentage of melanin release/content in comparison with α-MSH stimulated control cells (α-MSH+) (**A**,**B**) or as percentage change in viability in comparison to the vehicle (DMSO) treated control group (**C**). Each point represents the mean ± S.E.M. of three independent experiments performed in triplicates; # *p* < 0.001 vs. non-stimulated control cells (αMSH-); ** *p* < 0.01; *** *p* < 0.001 vs. αMSH+ cells.

To evaluate the effects of *F. penninervis* methanolic root extract and its isolated guaiane-type sesquiterpene lactones on the melanin synthesis and release, the melanin assay was carried out. For this purpose, the production of melanin in murine melanoma B16F10 cells was stimulated with αMSH. Indeed, the αMSH treatment induced a ~3fold increase in both melanin release in the conditioned medium and melanin content in the cell lysate as compared to non-treated cells (Figure 5A,B). Next, the treatment with the crude extract of *F. penninervis* (100 μg/mL) markedly decreased both the melanin release in the conditioned medium (to 26.97 ± 2.07% of αMSH+ control cells) and melanin content in the cell lysate (to 31.43 ± 8.47% of αMSH+ control cells, Figure 5A,B). In contrast to this, kojic acid (100 μg/mL), used as the positive control, only reduced the melanin release (35.88 ± 3.45% of αMSH+ control cells), but not the melanin content. Olgin, laferin, olgoferin and oferin significantly impaired the melanin release in the medium to 40.67 ± 5.75%–65.48 ± 2.12% of αMSH+ control cells; however, ferupennin Q and daucoguaianolactone F were found inactive. The following decreasing order of activity at 10 μg/mL was observed: olgoferin > laferin > olgin ~ oferin > daucoguaianolactone F > ferupennin Q. However, the melanin content was not significantly affected by any of the tested compounds. Our data could suggest that *Ferula* sesquiterpene lactones could primarily act as melanin release disrupting agents and not as melanin synthesis inhibitors. Furthermore, to exclude the possibility that the observed inhibitory effects on the melanin production might have resulted through a reduced number of viable cells, neutral red uptake assay at 72 h was performed (Figure 5C and Figure S17). Nevertheless, no treatment decreased the viability of B16F10 cells as compared to the negative control (αMSH-) cells. To our knowledge, there are no previous studies reporting the melanin production inhibitory potential of *Ferula* genus or isolated constituents. Nevertheless, the anti-melanogenic effects of different phytochemicals were repeatedly evidenced in various cell-based assays [46]. For instance, Mustapha et al. [45] showed that both the ethyl acetate leaf extract of *Crataegus azarolus* L. (12.5 μg/mL) and one of its main constituents, vitexin-2ʺ-*O*-rhamnoside (5 μM), decreased the production of intracellular melanin in B16F10 cells by threefold as compared to untreated cells. Measuring the melanin content and release in B16F10 cells was also used to prove the anti-melanogenic activity of piperlongumine derived from *Piper longum* L. [48]. Piperlongumine (3 and 6 μM) had no inhibitory effects on the cell growth, but it significantly reduced the total melanin production by down-regulating the tyrosinase activity and expression of several pro-melanogenic proteins, such as tyrosinase-related protein-1 (TRP-1), TRP2, and microphthalmia-associated transcription factor (MITF) [48].

## 3. Materials and Methods

### 3.1. Apparatus

A countercurrent chromatography (CCC) instrument (Dynamic Extractions, Slough, UK) equipped with analytical (0.8 mm i.d., 22 mL volume capacity, 1 mL injection loop) and semi-preparative (1.6 mm i.d., 136 mL volume capacity, 6 mL injection loop) polytetrafluoroethylene multilayer coils and connected to an Alpha 10 pump (ECOM, Prague, Czech Republic) and Sapphire UV-VIS detector (ECOM, Prague, Czech Republic) was used for the LLC separations. Semi-preparative HPLC experiments were performed on a Hitachi LaChrom 7000 HPLC system (Hitachi Ltd., Tokyo, Japan) equipped with a D-7000 interface, L-7150 pump, L-7420 DAD detector and Advantec SF-3120 fraction collector. Analytical HPLC-DAD analyses were carried out on a Shimadzu 20A series HPLC (Shimadzu, Tokyo, Japan) coupled with a DGU-20A 3R automatic degasser, LC-20AD quaternary pump, SIL-20A HT auto-sampler and SPD-M20A DAD detector. An Agilent 1200 HPLC system (Agilent Technologies, Santa Clara, CA, USA) equipped with a G1329A auto-sampler, G1379B degasser, G1312C binary pump, G1316B column oven, G1315B DAD detector and G6530B Q-TOF mass spectrometer was used for the HPLC-DAD-ESI-Q-TOF-MS investigations. A Bruker Avance Neo 600 MHz NMR spectrometer (Bruker BioSpin, Rheinstetten, Germany) supplied with a QCI 5 mm Cryoprobe and a SampleJet automated sample changer was employed for the NMR analyses. The cell morphology was examined using an inverted microscope (Nikon Eclipse, Nikon, Tokyo, Japan) and documented using an Invenio II camera (DeltaPix, Smorum, Denmark). The absorbance of the released neutral red was measured using FilterMax F5 microplate reader (Molecular Devices, San Jose, CA, USA).

### 3.2. Chemicals

Analytical grade ethyl acetate, methanol, and *n*-hexane were provided by POCh (Gliwice, Poland), while LC grade acetonitrile, methanol, formic acid and water were acquired from J. T. Baker (Deventer, The Netherlands). Deuterated methanol (CD_3_OD, 99.90% D) was bought from Eurisotop (Saarbrücken, Germany). 5-Fluorouracil (5-FU), α-melanocyte-stimulating hormone (αMSH), kojic acid, Dulbecco’s phosphate buffered saline (DPBS), 3.3 g/L neutral red solution in DPBS, Dulbecco′s Modified Eagle′s Medium (DMEM), DMEM:F12, Roswell Park Memorial Institute 1640 (RPMI-1640) medium, Ham’s F12 medium, and Eagle′s Minimum Essential Medium (EMEM) were purchased from Sigma-Aldrich (Darmstadt, Germany). Ethanol (>98%) and glacial acetic acid were purchased from Honeywell (Charlotte, NC, USA). Fetal bovine serum (FBS) was obtained from Pan-Biotech (Aidenbach, Germany).

### 3.3. Plant Material and Extraction

The roots of *Ferula penninervis* were collected from Almaty, Republic of Kazakhstan (GPS: 43.2147, 76.9121) in September 2018 by one of the authors (Z.S.) and authenticated by DrG.T. Sitbayeva from the Institute of Botany and Phytointroduction of the Committee for Science of the Ministry of Education and Science (Almaty, Republic of Kazakhstan). A voucher specimen (FP-01-07/300) was deposited in the Department of Pharmacognosy with Medicinal Plant Unit, Medical University of Lublin (Poland).

The air-dried and powdered roots of *F. penninervis* (50 g) were extracted with methanol (3 × 500 mL) by ultrasound assisted extraction (3 repeated cycles, each of 30 min), yielding (4.85 g, yield: 9.7%) of crude methanolic extract.

### 3.4. Isolation Procedures

#### 3.4.1. Liquid-Liquid Chromatographic Experiments

Several shake-flask experiments were initially performed in order to calculate the partition coefficient (Pi) values and select the optimum biphasic solvent system for LLC separations. Briefly, about 1 mg of crude extract was added to test tubes containing 4 mL of pre-equilibrated n-hexane/ethyl acetate/methanol/water (HEMWat) mixtures. The content was shaken for a full dissolution of the samples and left to stand until complete separation of the layers; afterwards, equal volumes (1 mL) of upper and lower phases were taken, evaporated to dryness, re-dissolved in 1 mL methanol and analyzed by HPLC-DAD. Next, the chosen biphasic solvent system was prepared in a separation funnel by serious shaking, equilibrated at room temperature, separated before use and degassed for 10 min.

The multilayer semi-preparative coil of the CCC unit was initially filled with the upper organic stationary phase. Then, the rotation of the apparatus was set to 1600 rpm and the lower aqueous mobile phase was pumped at a flow-rate of 6 mL/min (reversed-mode, head-to-tail). After system equilibration (attained when the volume of the displaced stationary phase was constant), 240 mg extract was dissolved in 3 mL upper phase and 3 mL lower phase and injected with a 6 mL loop; the effluent from the tail end of the column was monitored at 254 nm and one minute fractions were collected. The LLC separation was repeated three more times, under the above described conditions.

#### 3.4.2. Semi-Preparative HPLC Separations

Fractions that did not achieve a purity >90% after the LLC separations were further subjected to advanced purification by semi-preparative HPLC on a Cosmosil C18-AR-II (250 × 10 mm, 5 μm) column. The methanol concentrations used for the isocratic elution of the fractions were dependent on compounds polarity and were established through analytical HPLC-DAD experiments. The flow-rate (4 mL/min), injection volume (50 μL) and detection wavelength (254 nm) were kept constant.

#### 3.4.3. Analytical HPLC Separations

Partition coefficient values and purities of all LLC and semi-preparative HPLC collected fractions were checked by analytical HPLC-DAD experiments. Analyses were carried out on an Agilent Zorbax Eclipse XDB-C18 (250 × 4.6 mm, 5 μm) column, with water (A) and methanol (B) as mobile phases; the following elution gradient was applied: 50% B (0 min); 60% B (5 min); 80% B (25 min); 100% B (30–35 min); 1 mL/min flow-rate, 10 μL injection volume; 254 nm detection wavelength.

### 3.5. Structure Elucidation

All the isolated compounds were identified based on their UV, HRMS and NMR spectra. The HPLC-DAD-ESI-Q-TOF-MS analyses were performed on a Phenomenex Gemini C18 (100 × 2 mm, 3 µm) column, using 0.1% formic acid in water (A) and 0.1% formic acid in acetonitrile (B) with the following gradient: 15% B (0 min), 20% B (10 min), 40% B (45 min), 70% B (45 min), 90% B (46–50 min); 0.2 mL/min flow-rate; 5 μL injection volume; 200–400 nm DAD tracks. ESI-Q-TOF-MS data were recorded in positive mode; 100–1700 *m/z* mass range; 325 °C gas temperature; 12 L/min nitrogen flow; 30 psi nebulizer pressure; 65 V skimmer; 3500 V capillary voltage; 140 V fragmentor; 20 V collision energy. Full structural characterization was achieved in CD_3_OD by 1D NMR (^1^H-NMR; ^13^C-DEPTQ-NMR) and 2D NMR (correlation spectroscopy, COSY; heteronuclear multiple-bond correlation, HMBC; multiplicity-edited heteronuclear single-quantum correlation, edited-HSQC; rotating-frame Overhauser enhancement spectroscopy, ROESY) spectroscopic experiments.

**Compound 1**: UV (methanol) λ_max_ 254 nm; HREIMS *m/z* 389.1585 [M + H]^+^ (calcd for C_21_H_25_O_7_^+^, ∆ = 2.52 ppm); HRMS/MS(+) *m/z* 329.1385, 261.1155, 243.1080. ^1^H NMR (CD_3_OD, 600 MHz) δ 1.60 (3H, s, H-13), 1.98 (3H, t, *J* = 1.3 Hz, H-8d), 2.09 (3H, s, H-11b), 2.26 (6H, s, H-14, H-15), 2.68 (1H, dd, *J* = 19.1, 11.0 Hz, H-9′′), 2.91 (1H, dd, *J* = 19.1, 3.3 Hz, H-9′), 3.66 (1H, t, *J* = 11.0, 9.7 Hz, H-7), 3.96 (1H, d, *J* = 11.4 Hz, H-5), 4.70 (1H, t, *J* = 11.4, 9.7 Hz, H-6), 5.63 (1H, td, *J* = 11.0, 3.3 Hz, H-8), 5.74 (1H, p, *J* = 1.3 Hz, H-8c′′), 6.20 (2H, m, H-3, H-8c′); ^13^C NMR (CD_3_OD, 151 MHz) δ 18.3 (C-8d), 20.3 (C-14, C-15), 20.6 (C-13), 20.7 (C-11b), 44.6 (C-9), 48.7 (C-7), 48.9 (C-5), 69.5 (C-8), 79.5 (C-11), 80.2 (C-6), 127.3 (C-8c), 130.6 (C-1), 136.3 (C-3), 137.5 (C-8b), 147.7 (C-10), 167.5 (C-8a), 171.3 (C-11a), 173.1 (C-4), 175.6 (C-12), 197.6 (C-2).

**Compound 2**: UV (methanol) λ_max_ 254 nm; HREIMS *m/z* 391.1773 [M + H]^+^ (calcd for C_21_H_27_O_7_^+^, ∆ = –5.62 ppm); HRMS/MS(+) *m/z* 331.1561, 303.1242, 261.1134, 243.1031, 215.1072. ^1^H NMR (CD_3_OD, 600 MHz) δ 1.21 (3H, d, *J* = 7.0 Hz, H-8d), 1.24 (3H, d, *J* = 7.0 Hz, H-8c), 1.61 (3H, s, H-13), 2.10 (3H, s, H-11b), 2.25 (3H, s, H-14), 2.25 (3H, s, H-15), 2.61 (1H, hept, *J* = 7.0 Hz, H-8b), 2.65 (1H, dd, *J* = 19.1, 10.9 Hz, H-9′′), 2.79 (1H, dd, *J* = 19.1, 3.3 Hz, H-9′), 3.63 (1H, dd, *J* = 10.9, 9.7 Hz, H-7), 3.94 (1H, d, *J* = 11.4 Hz, H-5), 4.69 (1H, t, *J* = 11.4, 9.7 Hz, H-6), 5.60 (1H, td, *J* = 10.9, 3.3 Hz, H-8), 6.19 (1H, s, H-3); ^13^C NMR (CD_3_OD, 151 MHz) δ 19.0 (C-8d), 19.3 (C-8c), 20.2 (C-15), 20.3 (C-14), 20.7 (C-13), 20.7 (C-11b), 35.4 (C-8b), 44.4 (C-9), 48.5 (C-7), 48.8 (C-5), 68.7 (C-8), 79.4 (C-11), 80.2 (C-6), 130.6 (C-1), 136.3 (C-3), 147.7 (C-10), 171.3 (C-11a), 173.1 (C-4), 175.6 (C-12), 177.3 (C-8a), 197.6 (C-2).

**Compound 3**: UV (methanol) λ_max_ 253 nm; HREIMS *m/z* 403.1773 [M + H]^+^ (calcd for C_22_H_27_O_7_^+^, ∆ = –5.40 ppm); HRMS/MS(+) *m/z* 343.1609, 321.1403, 303.1287, 261.1174, 243.1065. ^1^H NMR (CD_3_OD, 600 MHz) δ 1.62 (3H, s, H-13), 1.94 (3H, p, *J* = 1.5 Hz, H-8e), 2.03 (3H, dq, *J* = 7.3, 1.5 Hz, H-8d), 2.07 (3H, s, H-11b), 2.25 (3H, s, H-14), 2.25 (3H, s, H-15), 2.68 (1H, dd, *J* = 19.1, 10.9 Hz, H-9′′), 2.88 (1H, dd, *J* = 19.1, 3.2 Hz, H-9′), 3.66 (1H, t, *J* = 10.9, 9.7 Hz, H-7), 3.95 (1H, d, *J* = 11.4 Hz, H-5), 4.69 (1H, dd, *J* = 11.4, 9.7 Hz, H-6), 5.66 (1H, td, *J* = 10.9, 3.2 Hz, H-8), 6.19 (1H, s, H-3), 6.26 (1H, qq, *J* = 7.3, 1.5 Hz, H-8c); ^13^C NMR (CD_3_OD, 151 MHz) δ 16.1 (C-8d), 20.3 (C-15), 20.3 (C-14), 20.6 (C-13), 20.6 (C-8e), 20.7 (C-11b), 44.6 (C-9), 48.7 (C-7), 48.9 (C-5), 68.7 (C-8), 79.5 (C-11), 80.3 (C-6), 128.3 (C-8b), 130.6 (C-1), 136.3 (C-3), 141.5 (C-8c), 147.8 (C-10), 167.8 (C-8a), 171.3 (C-11a), 173.1 (C-4), 175.6 (C-12), 197.6 (C-2).

**Compound 4**: UV (methanol) λ_max_ 253 nm; HREIMS *m/z* 415.1766 [M + H]^+^ (calcd for C_23_H_27_O_7_^+^, ∆ = –3.55 ppm); HRMS/MS(+) *m/z* 303.1351, 261.1238, 243.1120. ^1^H NMR (CD_3_OD, 600 MHz) δ 1.67 (3H, s, H-13), 1.94 (3H, t, *J* = 1.2 Hz, H-11d), 1.95 (3H, t, *J* = 1.3 Hz, H-8d), 2.25 (3H, s, H-14), 2.27 (3H, t, *J* = 1.4 Hz, H-15), 2.69 (1H, dd, *J* = 19.1, 10.8 Hz, H-9′′), 2.89 (1H, dd, *J* = 19.1, 3.3 Hz, H-9′), 3.71 (1H, dd, *J* = 11.2, 9.8 Hz, H-7), 3.99 (1H, d, *J* = 11.3 Hz, H-5), 4.75 (1H, dd, *J* = 11.3, 9.8 Hz, H-6), 5.69 (1H, td, *J* = 11.1, 3.3 Hz, H-8), 5.70 (1H, p, *J* = 1.3 Hz, H-8c′′), 5.75 (1H, p, *J* = 1.2 Hz, H-11c′′), 6.14 (1H, p, *J* = 1.3 Hz, H-8c′), 6.15 (1H, p, *J* = 1.2 Hz, H-11c′), 6.21 (1H, p, *J* = 1.4 Hz, H-3); ^13^C NMR (CD_3_OD, 151 MHz) δ 18.1 (C-11d), 18.3 (C-8d), 20.2 (C-15), 20.3 (C-14), 20.6 (C-13), 44.5 (C-9), 48.8 (C-5), 48.6 (C-7), 69.5 (C-8), 79.8 (C-11), 80.3 (C-6), 127.3 (C-8c), 128.0 (C-11c), 130.5 (C-1), 136.3 (C-3), 136.9 (C-11b), 137.5 (C-8b), 147.7 (C-10), 167.2 (C-11a), 167.4 (C-8a), 173.1 (C-4), 175.5 (C-12), 197.7 (C-2).

**Compound 5**: UV (methanol) λ_max_ 254 nm; HREIMS *m/z* 417.1926 [M + H]^+^ (calcd for C_23_H_29_O_7_^+^, ∆ = –4.37 ppm); HRMS/MS(+) *m/z* 347.1438, 261.1081, 243.0993, 225.0869. ^1^H NMR (CD_3_OD, 600 MHz) δ 1.17 (2H, d, *J* = 7.0 Hz, H-11d), 1.18 (5H, d, *J* = 7.0 Hz, H-11c), 1.62 (3H, s, H-13), 1.98 (3H, t, *J* = 1.3 Hz, H-8c), 2.25 (3H, s, H-14), 2.27 (3H, t, *J* = 1.4 Hz, H-15), 2.58 (1H, p, *J* = 7.0 Hz, H-11b), 2.69 (1H, dd, *J* = 19.1, 10.8 Hz, H-9′′), 2.89 (1H, dd, *J* = 19.1, 3.2 Hz, H-9′), 3.62 (1H, dd, *J* = 11.0, 9.7 Hz, H-7), 3.97 (1H, d, *J* = 11.4 Hz, H-5), 4.71 (1H, t, *J* = 11.4, 9.7 Hz, H-6), 5.66 (1H, td, *J* = 11.0, 3.2 Hz, H-8), 5.73 (1H, p, *J* = 1.3 Hz, H-8d′′), 6.18 (1H, p, *J* = 1.3 Hz, H-8d′), 6.20 (1H, q, *J* = 1.4 Hz, H-3); ^13^C NMR (CD_3_OD, 151 MHz) δ 18.3 (C-8c), 18.9 (C-11d), 19.1 (C-11c), 20.2 (C-14), 20.3 (C-15), 20.5 (C-13), 34.9 (C-11b), 44.5 (C-9), 48.9 (C-5), 49.0 (C-7), 69.6 (C-8), 79.4 (C-11), 80.3 (C-6), 127.2 (C-8d), 130.5 (C-1), 136.3 (C-3), 137.6 (C-8b), 147.8 (C-10), 167.5 (C-8a), 173.2 (C-4), 175.6 (C-12), 177.4 (C-11a), 197.7 (C-2).

**Compound 6**: UV (methanol) λ_max_ 254 nm; HREIMS *m/z* 419.2055 [M + H]^+^ (calcd for C_23_H_31_O_7_^+^, ∆ = 2.22 ppm); HRMS/MS(+) *m/z* 349.1549, 261.1034, 243.1055. ^1^H NMR (CD_3_OD, 600 MHz) δ 1.19 (3H, d, *J* = 6.9 Hz, H-11d), 1.20 (3H, d, *J* = 6.9 Hz, H-11c), 1.20 (3H, d, *J* = 6.9 Hz, H-8d), 1.23 (3H, d, *J* = 6.9 Hz, H- 8c), 1.61 (3H, s, H-13), 2.25 (3H, s, H-14), 2.26 (3H, t, *J* = 1.1 Hz, H-15), 2.60 (2H, hept, *J* = 6.9 Hz, H-8b, H-11b), 2.64 (1H, dd, *J* = 19.1, 10.8 Hz, H-9′′), 2.80 (1H, dd, *J* = 19.1, 3.3 Hz, H-9′), 3.55 (1H, dd, *J* = 11.1, 9.7 Hz, H-7), 3.94 (1H, d, *J* = 11.5 Hz, H-5), 4.70 (1H, dd, *J* = 11.5, 9.7 Hz, H-6), 5.60 (1H, td, *J* = 11.1, 3.3 Hz, H-8), 6.20 (1H, p, *J* = 1.1 Hz, H-3); ^13^C NMR (CD_3_OD, 151 MHz) δ 18.9 (C-11d), 19.0 (C-11c), 19.1 (C-8d), 19.3 (C-8c), 20.2 (C-15), 20.3 (C-14), 20.5 (C-13), 34.9 (C-11b), 35.4 (C-8b), 44.4 (C-9), 48.8 (C-7), 48.9 (C-5), 68.8 (C-8), 80.2 (C-6), 130.6 (C-1), 136.3 (C-3), 147.7 (C-10), 173.1 (C-4), 175.6 (C-12), 177.3 (C-11a), 177.3 (C-8a), 197.7 (C-2).

**Compound 7**: UV (methanol) λ_max_ 252 nm; HREIMS *m/z* 431.2062 [M + H]^+^ (calcd for C_24_H_31_O_7_^+^, ∆ = 0.53 ppm); HRMS/MS(+) *m/z* 361.1698, 343.1592, 331.1588, 261.1157, 243.1060, 225.0940. ^1^H NMR (CD_3_OD, 600 MHz) δ 1.15 (3H, d, *J* = 7.0 Hz, H-11d), 1.18 (3H, d, *J* = 7.0 Hz, H-11c), 1.63 (2H, s, H-13), 1.93 (3H, p, *J* = 1.6 Hz, H-8e), 2.01 (3H, dq, *J* = 7.3, 1.5 Hz, H-8d), 2.26 (3H, s, H-14), 2.27 (3H, t, *J* = 1.1 Hz, H-15), 2.56 (1H, hept, *J* = 7.0 Hz, H-11b), 2.70 (1H, dd, *J* = 19.0, 10.7 Hz, H-9′′), 2.88 (1H, dd, *J* = 19.0, 3.2 Hz, H-9′), 3.61 (1H, dd, *J* = 11.0, 9.7 Hz, H-7), 3.97 (1H, d, *J* = 11.4 Hz, H-5), 4.71 (1H, dd, *J* = 11.4, 9.7 Hz, H-6), 5.69 (1H, td, *J* = 10.9, 3.2 Hz, H-8), 6.20 (1H, p, *J* = 1.1 Hz, H-3), 6.22 (1H, qq, *J* = 7.3, 1.5 Hz, H-8c); ^13^C NMR (CD_3_OD, 151 MHz) δ 16.1 (C-8d), 18.7 (C-11d), 19.2 (C-11c), 20.3 (C-15), 20.3 (C-14), 20.5 (C-13), 20.7 (C-8e), 34.9 (C-11b), 44.7 (C-9), 48.9 (C-7), 48.9 (C-5), 68.7 (C-8), 79.4 (C-11), 80.4 (C-6), 128.5 (C-8b), 130.5 (C-1), 136.3 (C-3), 140.8 (C-8c), 147.9 (C-10), 167.9 (C-8a), 173.3 (C-4), 175.6 (C-12), 177.4 (C-11a), 197.7 (C-2).

### 3.6. Cell Viability Assay

#### 3.6.1. Cell Lines and Cell Cultures

Androgen-dependent prostate cancer (derived from lymph node metastasis) LNCaP (ATCC^®^ CRL-1740), androgen-insensitive prostate cancer (derived from brain metastasis) DU145 (ATCC^®^ HTB-81), androgen-insensitive prostate cancer (derived from bone metastasis) PC3 (ATCC^®^ CRL-1435) and prostate epithelial PNT2 (ECACC^®^ 95012613) cell lines were kindly provided by Dr. Vera Knäuper, School of Dentistry, Cardiff University (Cardiff, UK), whereas human colorectal carcinoma Caco-2 (ATCC^®^ HTB-37) cell line was provided by Dr. Konrad Szychowski (University of Information Technology and Management, Rzeszów, Poland). Human gastric cancer (derived from liver metastasis) N87 (ATCC^®^ CRL-5822), human colorectal cancer HT-29 (ATCC^®^ HTB-38), human malignant melanoma A375 (ATCC CRL-1619), human squamous cell cancer SCC-15 (ATCC^®^ CRL-1623) cell lines, murine melanoma B16F10 (ATCC^®^ CRL-6475) and human skin BJ fibroblast (ATCC^®^ CRL-2522) cell lines were purchased from LGC Standards (Łomianki, Poland), whilst immortalized human keratinocyte HaCaT cell lines were bought from Cell Lines Service (CLS) GmbH (Eppelheim, Germany).

LNCaP, PNT2 and N87 cells were grown in RPMI-1640 medium; PC3 cells were cultured in Ham’s F12; DU145, Caco-2 and BJ cells were maintained in EMEM. SCC-15 cell line was grown in DMEM:F12, whilst B16F10, HaCaT and HT-29 cell lines were maintained in DMEM. All conditioned media were supplemented with 10% FBS for all cell lines, except for Caco-2 cells which were grown in EMEM supplemented with 20% FBS. The cells were cultured at 37 °C in a humidified atmosphere with 5% CO_2_.

#### 3.6.2. Neutral Red Uptake Assay

Neutral red uptake (NRU) assay was performed as described by Repetto et al. [49]. Briefly, cells were seeded in 96-well plates (3 × 10^3^ cells/well) and grown overnight. The cells were then treated with increasing concentrations of *F. penninervis* crude extract (12.5–200 µg/mL) and isolated compounds (3.125–50 µg/mL) or the positive control 5-FU (1 µg/mL). The control cells were grown in appropriate culture medium containing equal volume of the used solvent (DMSO). After 48 h, the cells were incubated for 3 h with neutral red solution (33 µg/mL) in conditioned medium containing 1% FBS. The morphology of the cells was examined with an inverted microscope and documented using an Invenio II camera. The cells were then rinsed with DPBS and lysed using acidified ethanol solution (50% *v/v* ethanol, 1% *v/v* acetic acid and 49% H_2_O). The absorbance of the released neutral red was measured at λ = 540 nm using FilterMax F5 microplate reader and corrected by the absorbance at λ = 620 nm. The mean measured value for the lysate from control cells was set as 100% cellular viability and used to calculate the percentage of viable cells following extracts treatment.

### 3.7. Melanin Release/Content Assay

B16F10 cells were plated in 6-well plates (0.5 × 10^5^ cells/well) and grown overnight. The cells were treated with *F. penninervis* crude extract (100 µg/mL), isolated compounds (10 µg/mL) or the positive control kojic acid (100 μg/mL). Melanin production was stimulated with α-MSH (10 nM). Following 72 h, the conditioned medium and cell pellets were collected. Cell pellets were dissolved in 1 N NaOH and incubated for 2 h at 80 °C. Conditioned media and cell lysates were then transferred to a 96-well plate and the absorbance was measured at λ = 405 nm using FilterMax F5 microplate reader. The content of protein in cell lysates was establish using Bradford assay [50]. The melanin released in the conditioned medium and the melanin content in cell lysates (μg melanin/mg protein) were calculated with the help synthetic melanin calibration curves. The melanin release/content was expressed as the percentage of melanin release/content in comparison with α-MSH stimulated control cells without the tested samples.

### 3.8. Statistical Analysis

All experiments were performed in triplicate and the results were expressed as mean ± standard error of mean (SEM). Data are representative of at least three individual experiments. Statistical analysis was performed with OriginPro 2020 (OriginLab, Northampton, MA, USA) using one-way analysis of variance (ANOVA) with Turkey’s post-host test; *p* < 0.05 was considered statistically significant. The concentrations required to reduce the cell viability percentages to 50% (IC_50_) were obtained by non-linear regression analysis using quick fit-dose response (sigmoidal fit with Boltzmann function).

## 4. Conclusions

In this study, two new (penninervins P and Q) and five known (olgin, laferin, olgoferin, oferin and daucoguainolactone F) guaiane-type sesquiterpene lactones were isolated from *F. penninervis* methanolic root extract with the use of a simple and fast LLC method. None of the isolated compounds were previously separated by LLC from the *Ferula* genus. Among the sesquiterpene lactones, olgin and laferin showed the most promising cytotoxic effects in androgen-dependent prostate cancer LNCaP cells. Furthermore, up to the highest tested concentration (50 μg/mL), olgin did not significantly affect the viability of the non-cancer prostate epithelial PNT2 control cells, indicating a potential cancer cell selectivity. Additionally, olgin, laferin, olgoferin and oferin potently down-regulated the melanin release in the conditioned medium of αMSH-stimulated murine melanoma B16F10 cells without influencing their cellular survival rates. Overall, the results of this study suggest that a few guaiane-type sesquiterpene lactones isolated from *F. penninervis* root extract (e.g., olgin and laferin) might be endowed with anti-prostate cancer and skin depigmentation properties. However, further investigations are needed to get more evidence for the potential clinical use of these phytochemicals in cancer chemotherapy or hyperpigmentation disorders.

## Figures and Tables

**Table 1:** Partition coefficient values of the target compounds from *Ferula penninervis* methanolic root extract.

No.	HEMWat	*P*_1_	*P*_2_	*P*_3_	*P*_4_	*P*_5_	*P*_6_	*P*_7_
1	5/6/5/6	5.20	5.28	8.18	10.13	14.20	15.30	21.77
2	1/1/1/1	1.91	2.04	3.03	4.69	5.34	5.48	7.95
3	6/5/6/5	0.95	1.04	1.66	2.43	2.89	2.98	4.49
4	3/2/3/2	0.44	0.48	0.70	1.02	1.27	1.32	1.89
	*α*	*−*	*1.1*	*1.5*	*1.5*	*1.3*	*1.0*	*1.4*

α separation factor; HEMWat *n*-hexane/ethyl acetate/methanol/water; *P* partition coefficient; with bold the selected biphasic solvent system for the liquid-liquid chromatography experiments.

**Table 2:** ^1^H NMR (600 MHz) and ^13^C NMR (151 MHz) data of ferupennin P (**2**) and ferupennin Q (**6**) in CD_3_OD; δ in ppm; *J* in Hz.

Position	Ferupennin P (2)		Ferupennin Q (6)	
	δ_H_ (Multiplicity, *J*, nH)	δ_C_	δ_H_ (Multiplicity, *J*, nH)	δ_C_
*Sesq.*				
1		130.6		130.6
2		197.6		197.7
3	6.19 (s, 1H)	136.3	6.20 (p, 1.1 Hz, 1H)	136.3
4		173.1		173.1
5	3.94 (d, 11.4 Hz, 1H)	48.8	3.94 (d, 11.5 Hz, 1H)	48.9
6	4.69 (t, 11.4, 9.7 Hz, 1H)	80.2	4.70 (dd, 11.5, 9.7 Hz, 1H)	80.2
7	3.63 (dd, 10.9, 9.7 Hz, 1H)	48.5	3.55 (dd, 11.1, 9.7 Hz, 1H)	48.8
8	5.60 (td, 10.9, 3.3 Hz, 1H)	68.7	5.60 (td, 11.1, 3.3 Hz, 1H)	68.8
9	2.79 (dd, 19.1, 3.3 Hz, 1H) 2.65 (dd, 19.1, 10.9 Hz, 1H)	44.4	2.80 (dd, 19.1, 3.3 Hz, 1H) 2.64 (dd, 19.1, 10.8 Hz, 1H)	44.4
10		147.7		147.7
11		79.4		79.3
12		175.6		175.6
13	1.61 (s, 3H)	20.7	1.61 (s, 3H)	20.5
14	2.25 (s, 3H)	20.3	2.25 (s, 3H)	20.3
15	2.25 (s, 3H)	20.2	2.26 (t, 1.1 Hz, 3H)	20.2
*Acyl-8*				
8a		177.3		177.3
8b	2.61 (hept, 7.0 Hz, 1H)	35.4	2.60 (hept, 6.9 Hz, 1H)	35.4
8c	1.24 (d, 7.0 Hz, 3H)	19.3	1.23 (d, 6.9 Hz, 3H)	19.3
8d	1.21 (d, 7.0 Hz, 3H)	19.0	1.20 (d, 6.9 Hz, 3H)	19.1
*Acyl-11*				
11a		171.3		177.3
11b	2.10 (s, 3H)	20.7	2.60 (hept, 6.9 Hz, 1H)	34.9
11c			1.20 (d, 6.9 Hz, 3H)	19.0
11d			1.19 (d, 6.9 Hz, 3H)	18.9

**Table 3:** Cytotoxic effects of Ferula *penninervis* methanolic root extract against gastrointestinal cancer (N87, Caco-2, HT-29) cells, prostate cancer cells (LNCaP, DU145, PC3), skin cancer cells (SCC-15, A375, B16F10) and non-cancer cells (PNT2, HaCaT, BJ); data represent the mean ± S.E.M. of three independent experiments performed in triplicates.

Cell Line-Type	Cell Line	IC_50_ (μg/mL)
Gastrointestinal cancer cells	N87	154.54 ± 4.13
	Caco-2	145.08 ± 6.82
	HT-29	190.53 ± 9.90
Prostate cancer cells	LNCaP	112.12 ± 3.82
	DU145	>200
	PC3	112.28 ± 3.98
Skin cancer cells	SCC-15	77.84 ± 2.55
	A375	138.48 ± 9.45
	B16F10	110.04 ± 1.34
Non-cancer cells	PNT2	>200
	HaCaT	159.28 ± 5.70
	BJ	>200

## Data Availability

Not applicable.

## Supplementary Materials

The following are available online at https://www.mdpi.com/article/10.3390/ijms221910717/s1.
