# Supplementary material for: Liquid-Liquid Chromatography Separation of Guaiane-Type Sesquiterpene Lactones from Ferula penninervis Regel & Schmalh. and Evaluation of Their In Vitro Cytotoxic and Melanin Inhibitory Potential
Source: Int J Mol Sci. 2021 Oct 3;22(19):10717. doi: 10.3390/ijms221910717 (PMC8509705; doi:10.3390/ijms221910717)
Supplement: Supplementary file 1 [file ijms-22-10717-s001.zip › ijms-1368497-supplementary.pdf]

Supplementary Material

Liquid-liquid Chromatography Separation of Guaiane-Type Sesquiterpene Lactones from *Ferula penninervis* Regel & Schmalh. and Evaluation of Their *in vitro* Cytotoxic and Melanin Inhibitory Potential

Simon Vlad Luca [1,2,] *, Katarzyna Gaweł-Bęben [3,], Marcelina Strzępek-Gomółka[3], Ainur Jumabayeva[4], Zuriyadda Sakipova[4], Jianbo Xiao[5], Laurence Marcourt[6,7], Jean-Luc Wolfender[6,7], Krystyna Skalicka-Woźniak[8]**

[1] Biothermodynamics, TUM School of Life Sciences, Technical University of Munich, 85354 Freising, Germany
[2] Department of Pharmacognosy, "Grigore T. Popa" University of Medicine and Pharmacy Iasi, 700115 Iasi, Romania
[3] Department of Cosmetology, University of Information Technology and Management in Rzeszów, 35-225 Rzeszów, Poland
[4] School of Pharmacy, Kazakh National Medical University named after S.D. Asfendiyarov (KazNMU), Almaty 050012, Republic of Kazakhstan
[5] Nutrition and Bromatology Group, Department of Analytical Chemistry and Food Science, Faculty of Food Science and Technology, University of Vigo - Ourense Campus, Ourense, E-32004, Spain
[6] School of Pharmaceutical Sciences, University of Geneva, CMU - Rue Michel-Servet 1, CH-1211 Geneva 4, Switzerland
[7] Institute of Pharmaceutical Sciences of Western Switzerland, IPSWS, University of Geneva, CMU, 1211 Geneva 4, Switzerland
[8] Independent Laboratory of Natural Products Chemistry, Medical University of Lublin, 20-093 Lublin, Poland
* Correspondence: vlad.luca@tum.de (V.L.); kagawel@wsiz.edu.pl (K.G.B.)

Supplementary file content

Part A. Isolation studies

Fig. S1. HPLC-DAD chromatograms of isolated compounds from *Ferula penninervis*

Part B. Spectroscopic data of new isolated compounds

PartB1. Spectroscopic data of Ferupennin P (**2**)

Fig. S2. HRMS/MS spectrum of Ferupennin P (**2**)

Fig. S3. ^{1}H NMR spectrum of Ferupennin P (**2**) in CD_3OD at 600 MHz

Fig. S3a. ^{1}H NMR spectrum of Ferupennin P (2) in CD3OD at 600 MHz Zoom 1

Fig. S3b. ^{1}H NMR spectrum of Ferupennin P (2) in CD3OD at 600 MHz Zoom 2

Fig. S4. COSY NMR spectrum of Ferupennin P (**2**) **A2** in CD_3OD

Fig. S5. ^{13}C-DEPTQ NMR spectrum of Ferupennin P (**2**) in CD_3OD at 151 MHz

Fig. S6. Edited-HSQC NMR spectrum of Ferupennin P (**2**) in CD_3OD

Fig. S7. HMBC NMR spectrum of Ferupennin P (**2**) in CD_3OD

Fig. S8. ROESY NMR spectrum of Ferupennin P (**2**) in CD_3OD

PartB2. Spectroscopic data of Ferupennin Q (**6**)

Fig. S9. HRMS/MS spectrum of Ferupennin Q (**6**)

Fig. S10. ^{1}H NMR spectrum of Ferupennin Q (**6**) in CD_3OD at 600 MHz

Fig. S10a. ^{1}H NMR spectrum of Ferupennin Q (**6**) in CD3OD at 600 MHz Zoom 1

Fig. S10b. ^{1}H NMR spectrum of Ferupennin Q (**6**) in CD3OD at 600 MHz Zoom 2

Fig. S11. COSY NMR spectrum of Ferupennin Q (**6**) in CD_3OD

Fig. S12. ^{13}C-DEPTQ NMR spectrum of Ferupennin Q (**6**) in CD_3OD at 151 MHz

Fig. S13. Edited-HSQC NMR spectrum of Ferupennin Q (**6**) in CD_3OD

Fig. S14. ROESY NMR spectrum of Ferupennin Q (**6**) in CD_3OD

Part C. Cytotoxicity evaluations

Fig. S15. Morphology of prostate epithelial cells PNT2 and prostate cancer cell lines LNCaP, DU145, PC3 cells grown for 48h with various concentrations of *F. penninervis* root extract, DMSO as a solvent control or 5-fluorouracil (5 µg/mL); neutral red staining, 10 × magnification; pictures are representative for 3 experiments

Fig. S16. Morphology of LNCaP prostate cancer cells grown for 48h with various concentrations of F. penninervis compounds or DMSO as a solvent control; neutral red staining, 10 × magnification; pictures are representative for 3 experiments

Fig. S17. Morphology of murine melanome B16F10 cells grown for 72 h with DMSO (untreated), αMSH (10 nM) and *F. penninervis* root extract (100 µg/mL), kojic acid (100 µg/mL) or isolated sesquiterpene lactones (10 µg/mL); neutral red staining, 10 × magnification; pictures are representative for 3 experiments

Part A. Isolation studies

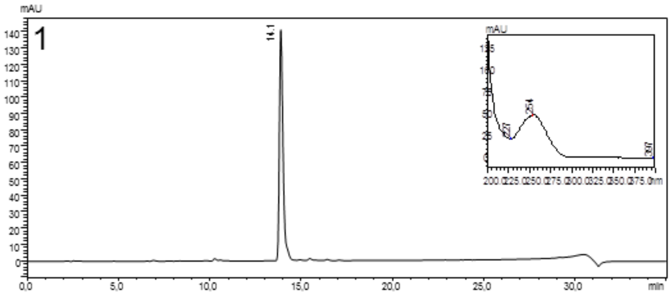

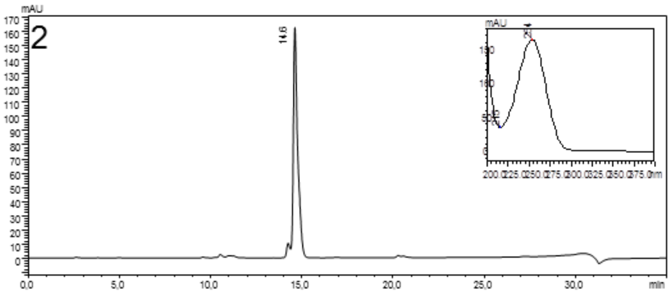

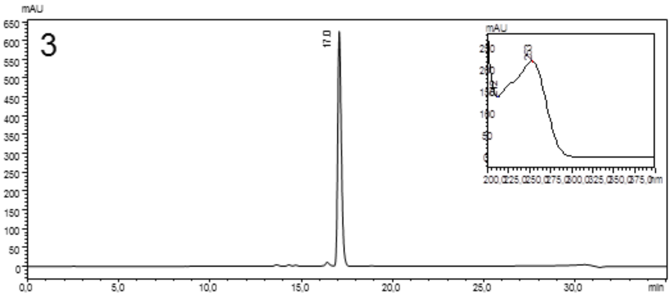

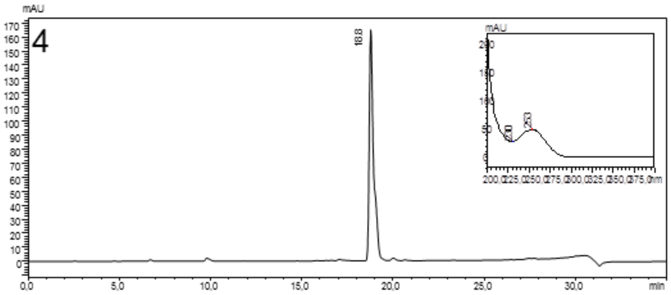

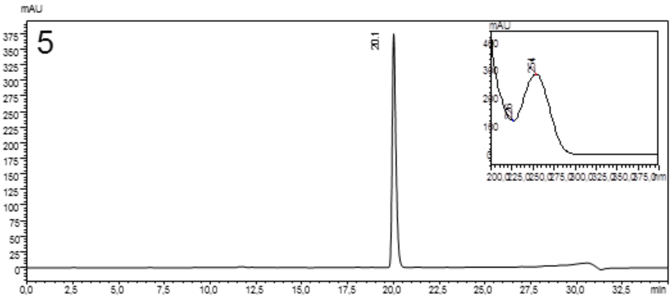

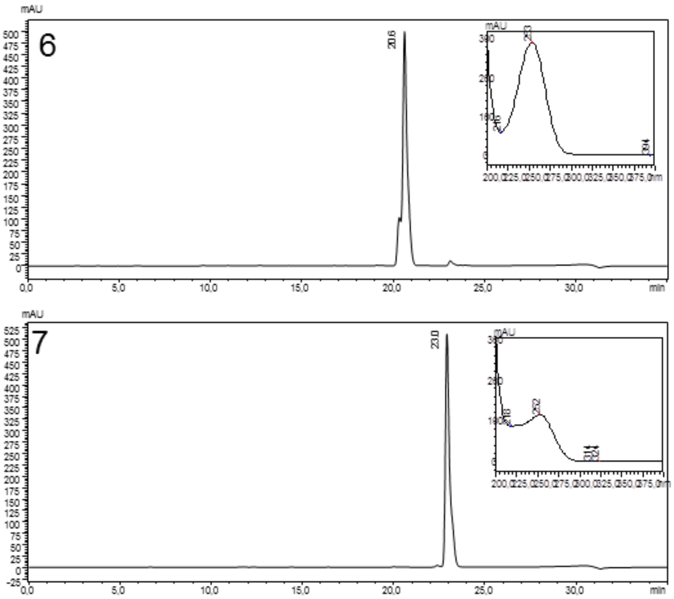

Fig. S1. HPLC-DAD chromatograms and UV spectra of isolated compounds from *Ferula penninervis* methanolic root extract

Column: Agilent Zorbax Eclipse XDB-C18 (250 × 4.6 mm, 5 μm); Mobile phase: water (A) and methanol (B); Gradient: 50% B (0 min); 60% B (5 min); 80% B (25 min); 100% B (30–35 min); Flow-rate: 1 mL/min; UV: 254 nm

Part B. Spectroscopic data of new isolated compounds

***PartB1*. Spectroscopic data of Ferupennin P (2)**

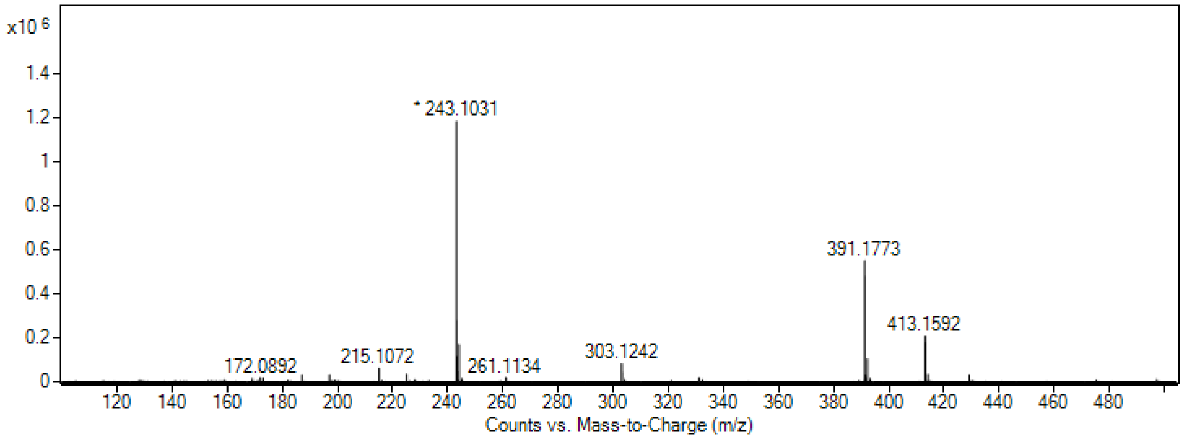

Fig. S2. HRMS/MS spectrum of Ferupennin P (**2**)

^{1}*H NMR* (CD_3OD, 600 MHz) δ 1.21 (3H, d, *J*=7.0 Hz, H-8d), 1.24 (3H, d, *J*=7.0 Hz, H-8c), 1.61 (3H, s, H-13), 2.10 (3H, s, H-11b), 2.25 (3H, s, H-14), 2.25 (3H, s, H-15), 2.61 (1H, hept, *J*=7.0 Hz, H-8b), 2.65 (1H, dd, *J*=19.1, 10.9 Hz, H-9''), 2.79 (1H, dd, *J*=19.1, 3.3 Hz, H-9'), 3.63 (1H, dd, *J*=10.9, 9.7 Hz, H-7), 3.94 (1H, d, *J*=11.4 Hz, H-5), 4.69 (1H, t, *J*=11.4, 9.7 Hz, H-6), 5.60 (1H, td, *J*=10.9, 3.3 Hz, H-8), 6.19 (1H, s, H-3);
^{13}*C NMR* (CD_3OD, 151 MHz) δ 19.0 (C-8d), 19.3 (C-8c), 20.2 (C-15), 20.3 (C-14), 20.7 (C-13), 20.7 (C-11b), 35.4 (C-8b), 44.4 (C-9), 48.5 (C-7), 48.8 (C-5), 68.7 (C-8), 79.4 (C-11), 80.2 (C-6), 130.6 (C-1), 136.3 (C-3), 147.7 (C-10), 171.3 (C-11a), 173.1 (C-4), 175.6 (C-12), 177.3 (C-8a), 197.6 (C-2)

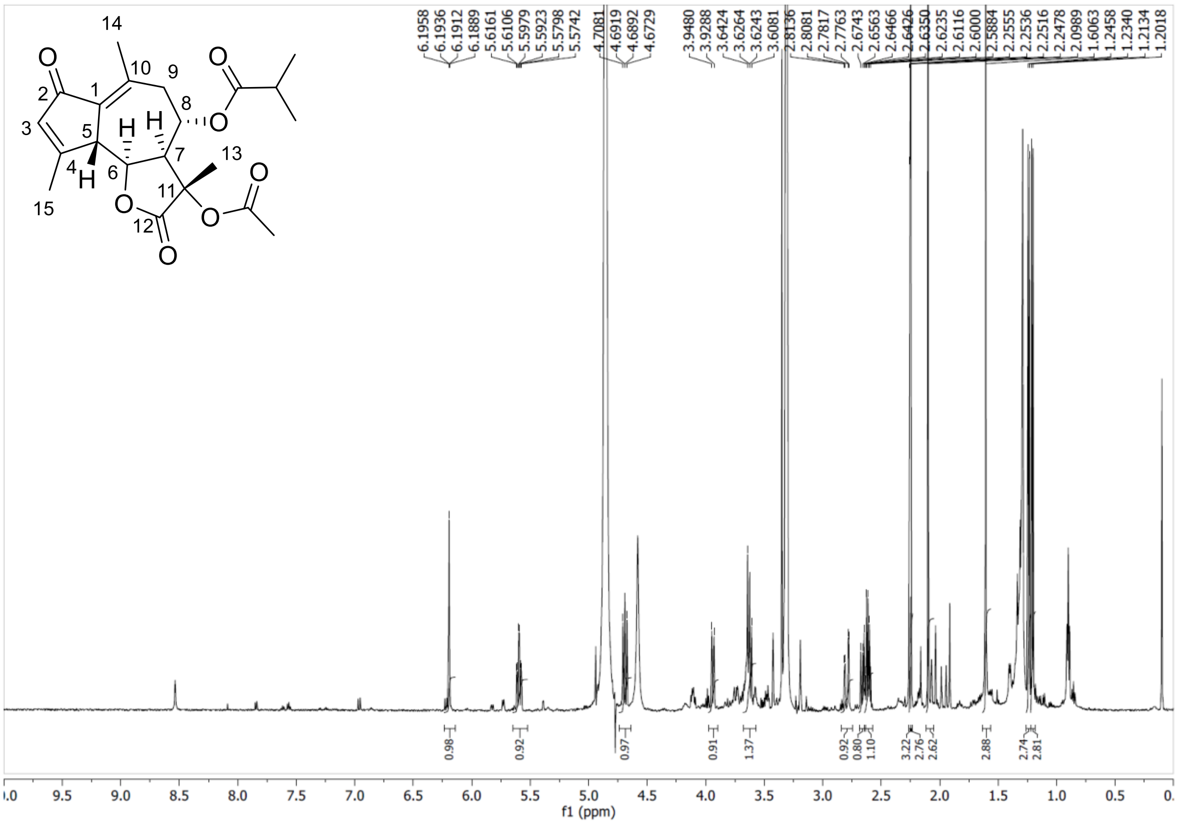

Fig. S3. ^{1}H NMR spectrum of Ferupennin P (**2**) in CD_3OD at 600 MHz

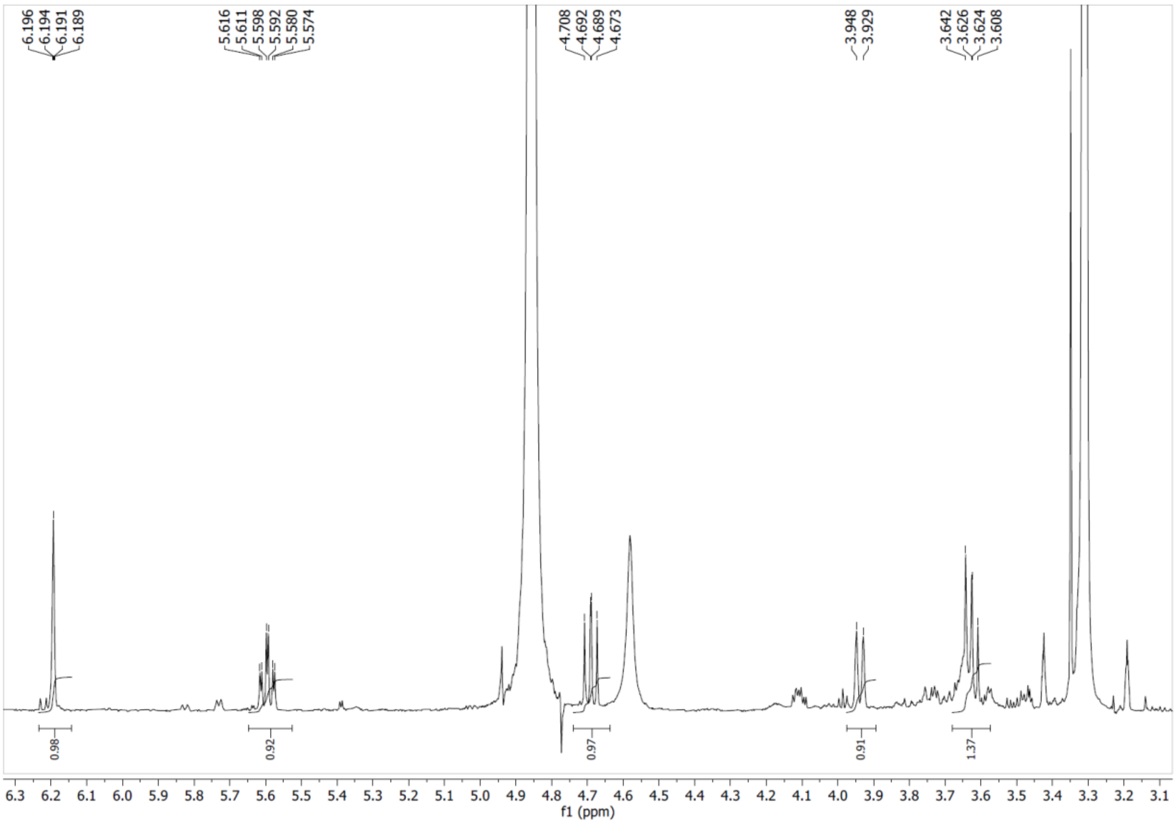

Fig. S3a. ^{1}H NMR spectrum of Ferupennin P (**2**) in CD_3OD at 600 MHz Zoom 1

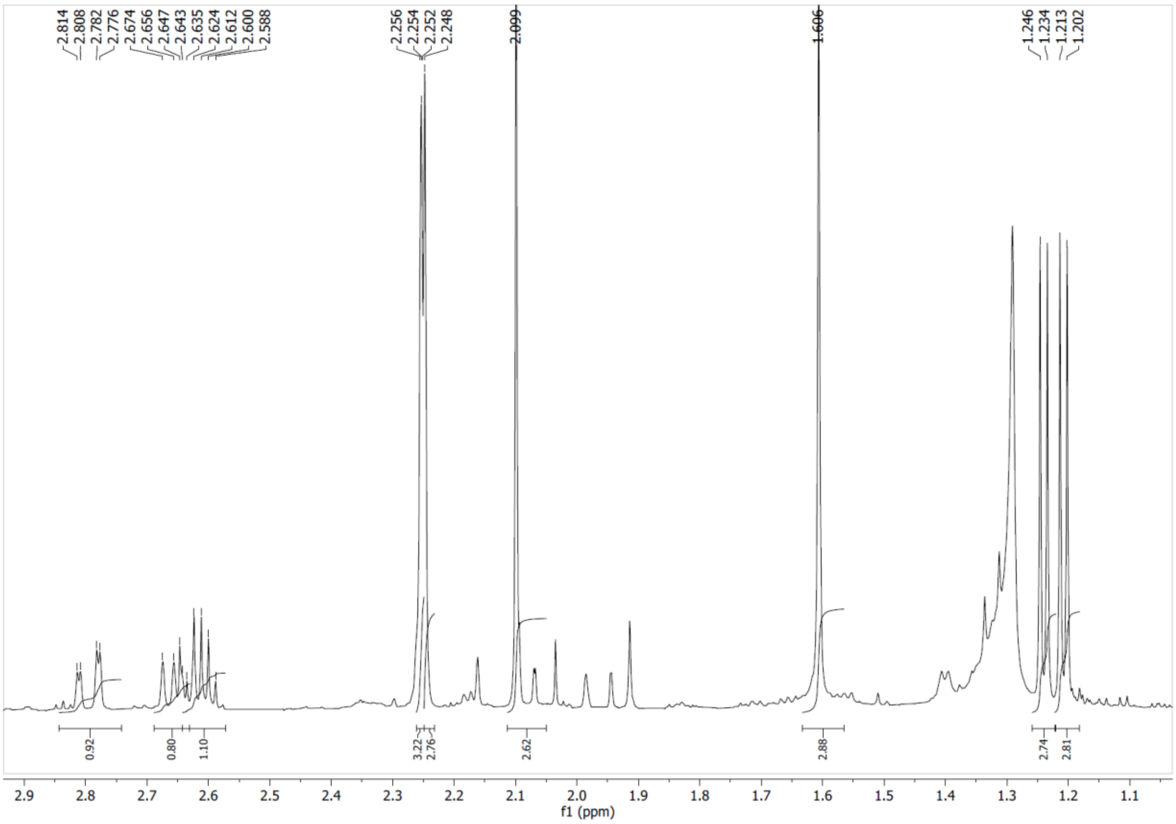

Fig. S3b. ^{1}H NMR spectrum of Ferupennin P (**2**) in CD3OD at 600 MHz Zoom 2

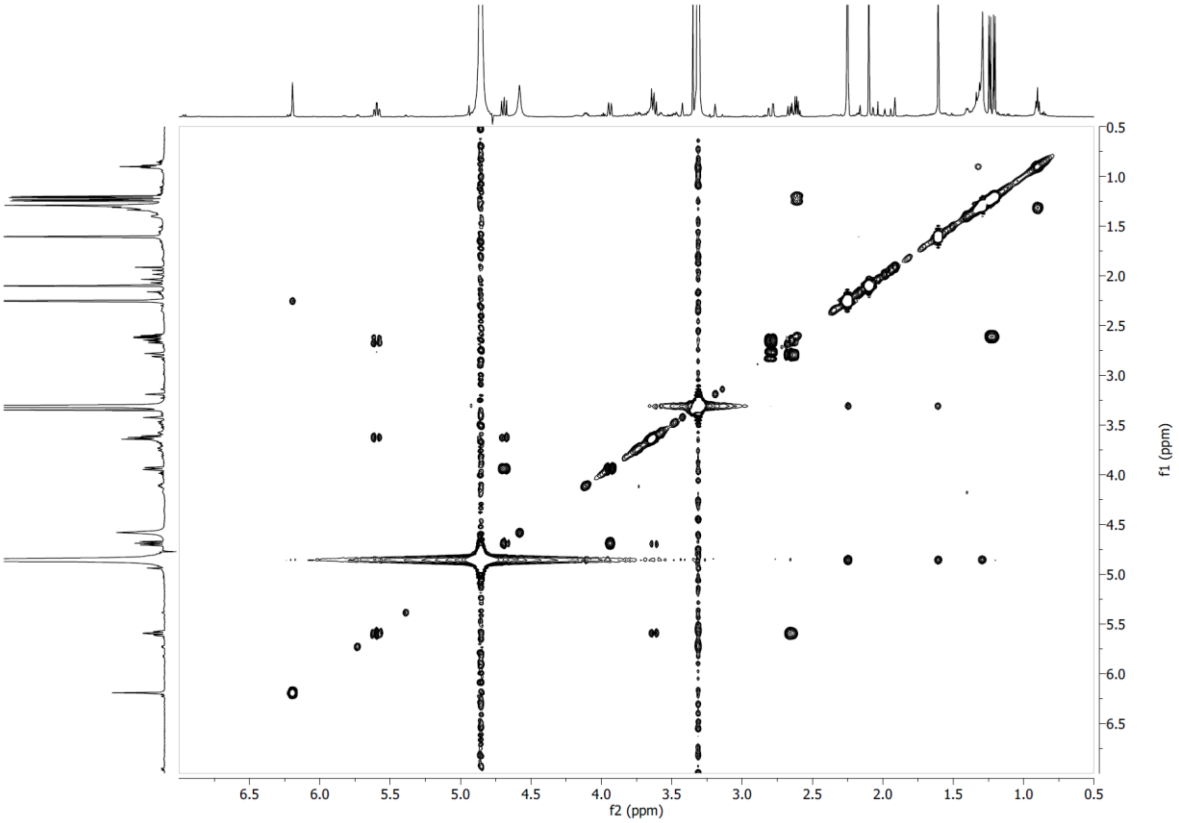

Fig. S4. COSY NMR spectrum of Ferupennin P (**2**) **A2** in CD_3OD

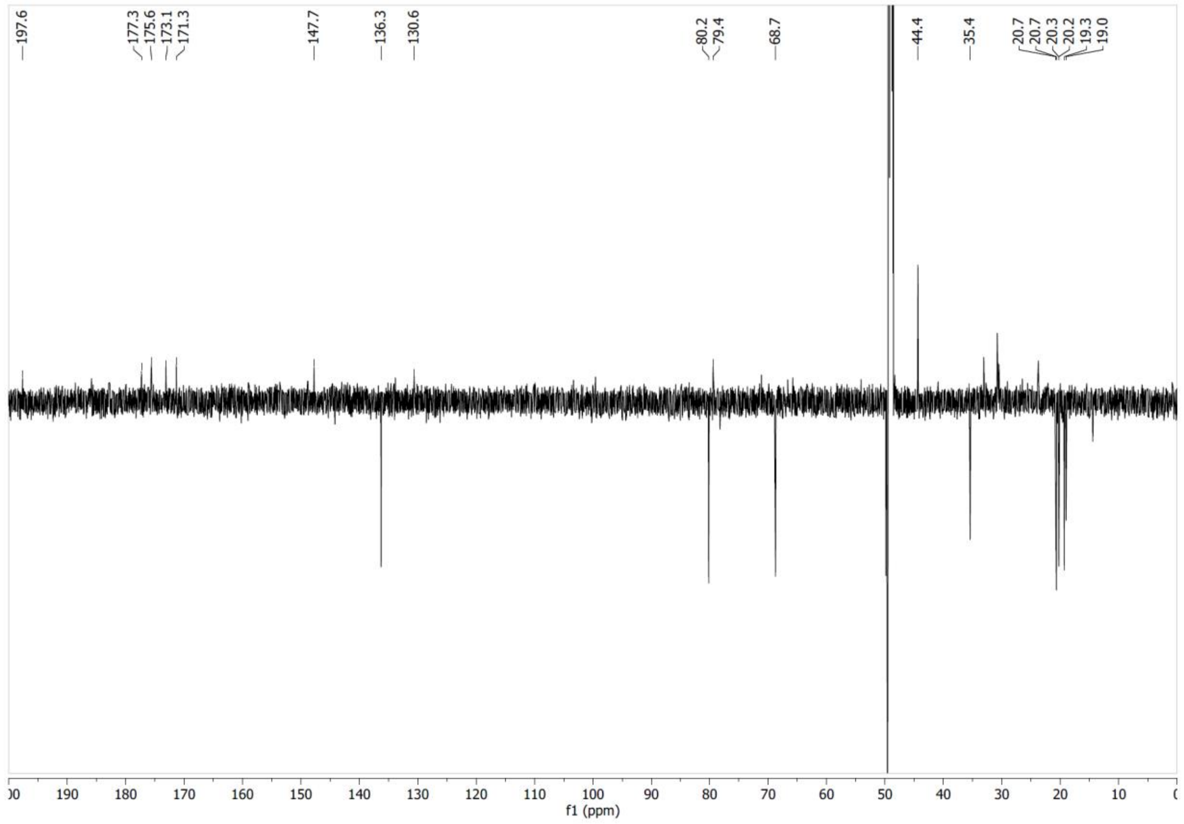

Fig. S5. ^{13}C-DEPTQ NMR spectrum of Ferupennin P (**2**) in CD_3OD at 151 MHz

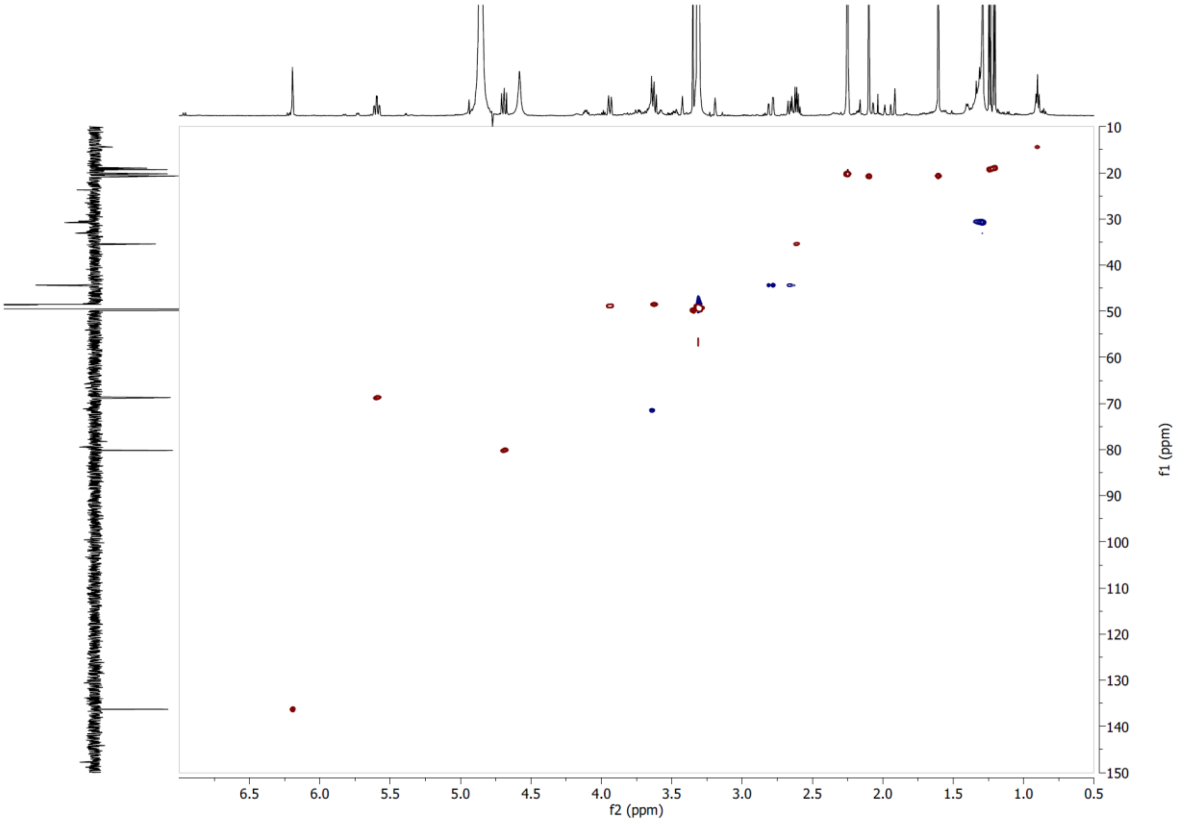

Fig. S6. Edited-HSQC NMR spectrum of Ferupennin P (**2**) in CD_3OD

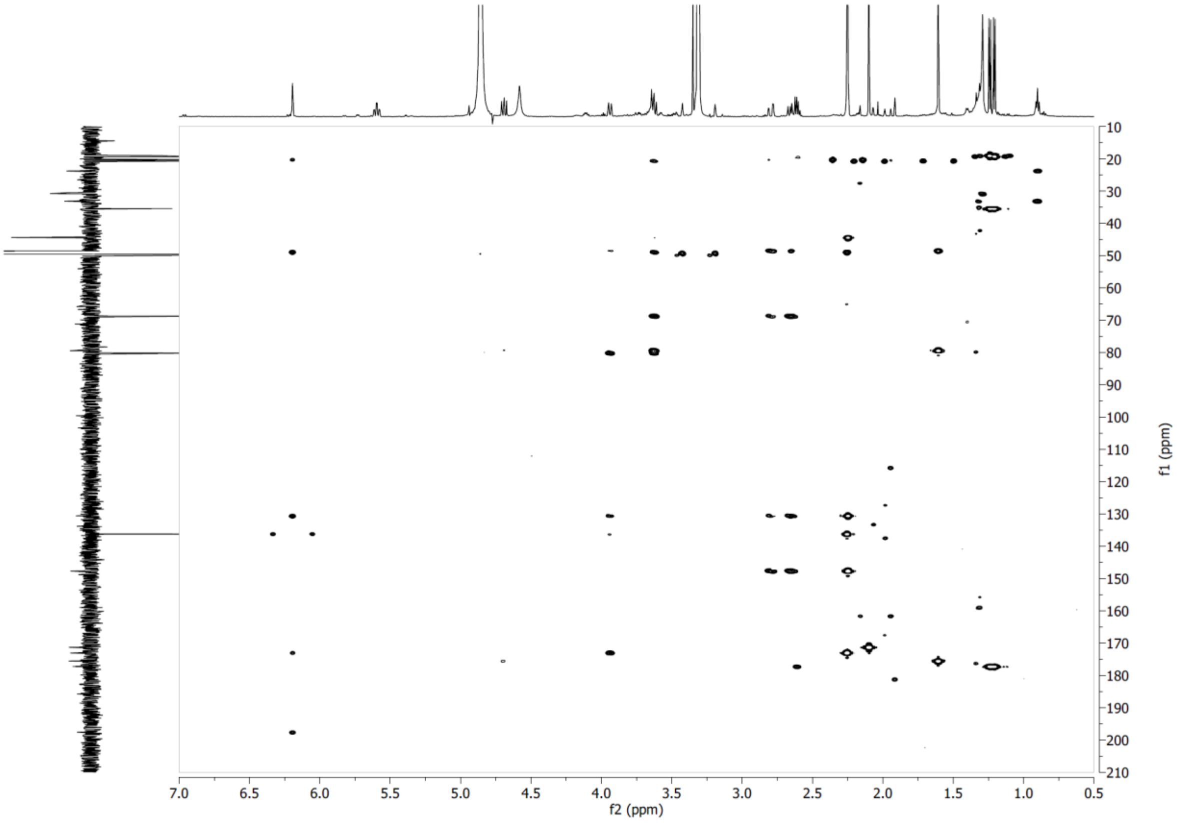

Fig. S7. HMBC NMR spectrum of Ferupennin P (**2**) in CD_3OD

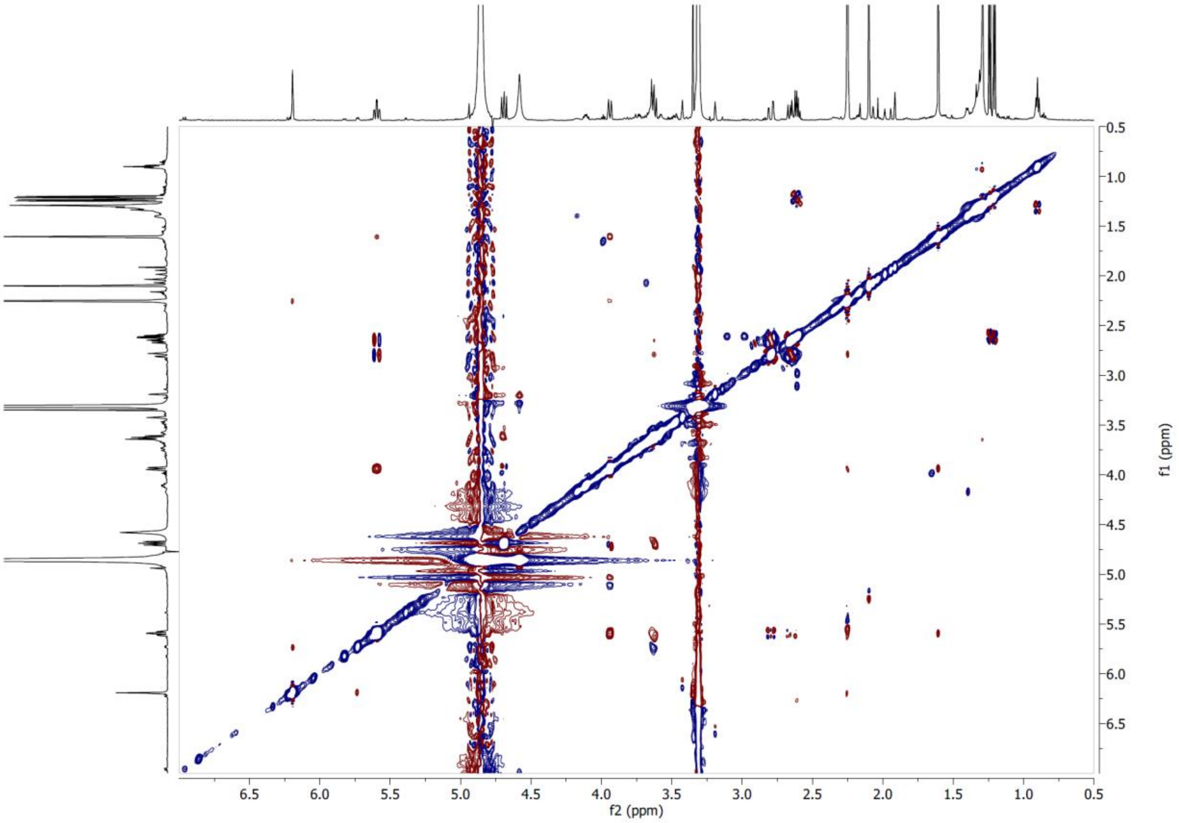

Fig. S8. ROESY NMR spectrum of Ferupennin P (**2**) in CD_3OD

***PartB2*. Spectroscopic data of Ferupennin Q (6)**

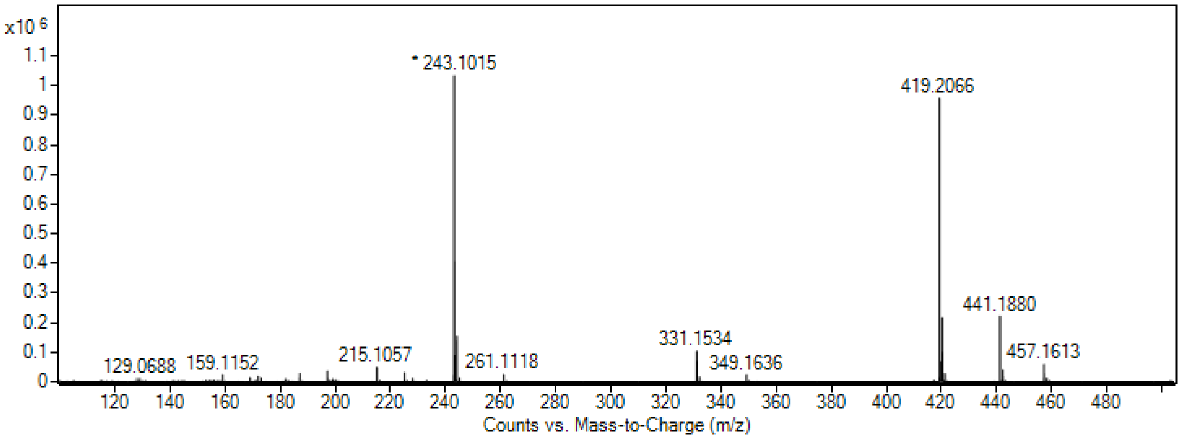

Fig. S9. HRMS/MS spectrum of Ferupennin Q (**6**)

^{1}H NMR (CD_3OD, 600 MHz) δ 1.19 (3H, d, *J*=6.9 Hz, H-11d), 1.20 (3H, d, *J*=6.9 Hz, H-11c), 1.20 (3H, d, *J*=6.9 Hz, H-8d), 1.23 (3H, d, *J*=6.9 Hz, H- 8c), 1.61 (3H, s, H-13), 2.25 (3H, s, H-14), 2.26 (3H, t, *J*=1.1 Hz, H-15), 2.60 (2H, hept, *J*=6.9 Hz, H-8b, H-11b), 2.64 (1H, dd, *J*=19.1, 10.8 Hz, H-9''), 2.80 (1H, dd, *J*=19.1, 3.3 Hz, H-9'), 3.55 (1H, dd, *J*=11.1, 9.7 Hz, H-7), 3.94 (1H, d, *J*=11.5 Hz, H-5), 4.70 (1H, dd, *J*=11.5, 9.7 Hz, H-6), 5.60 (1H, td, *J*=11.1, 3.3 Hz, H-8), 6.20 (1H, p, *J*=1.1 Hz, H-3)
^{13}C NMR (CD_3OD, 151 MHz) δ 18.9 (C-11d), 19.0 (C-11c), 19.1 (C-8d), 19.3 (C-8c), 20.2 (C-15), 20.3 (C-14), 20.5 (C-13), 34.9 (C-11b), 35.4 (C-8b), 44.4 (C-9), 48.8 (C-7), 48.9 (C-5), 68.8 (C-8), 79.3 (C-11), 80.2 (C-6), 130.6 (C-1), 136.3 (C-3), 147.7 (C-10), 173.1 (C-4), 175.6 (C-12), 177.3 (C-11a), 177.3 (C-8a), 197.7 (C-2)

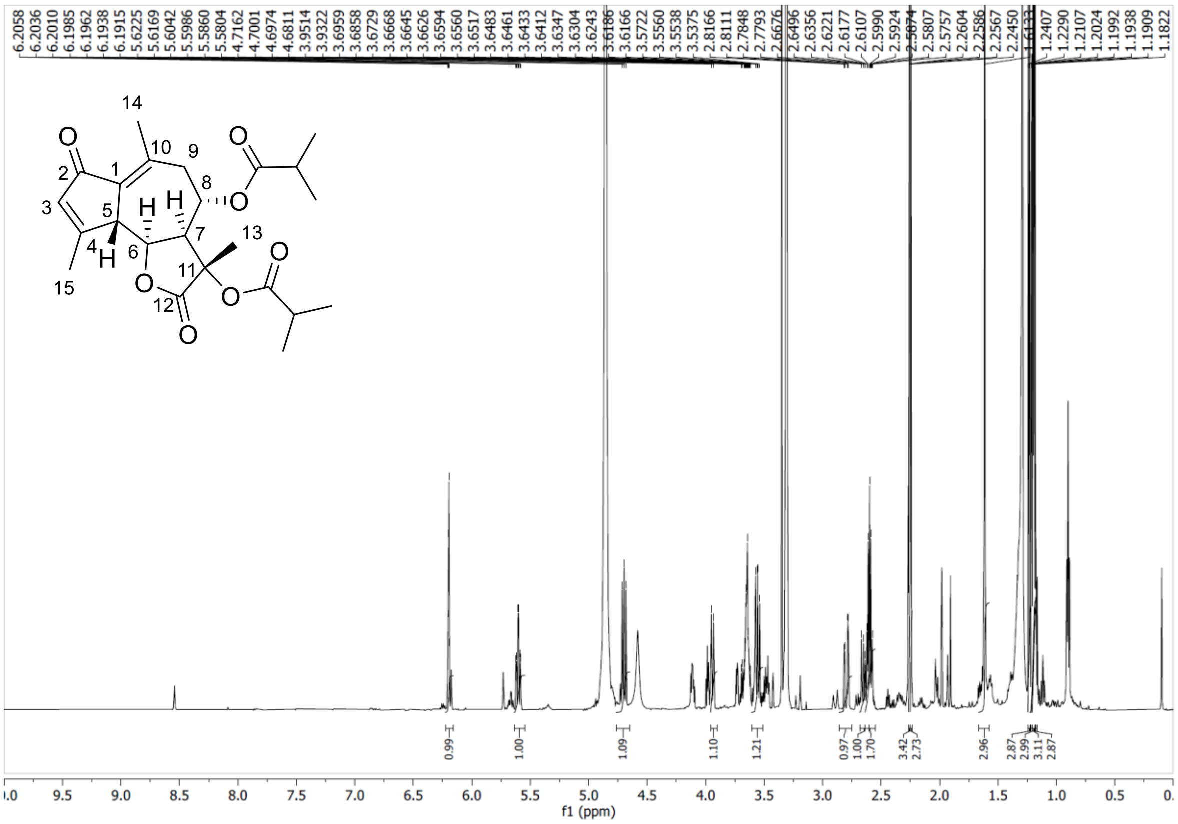

Fig. S10. ^{1}H NMR spectrum of Ferupennin Q (**6**) in CD_3OD at 600 MHz

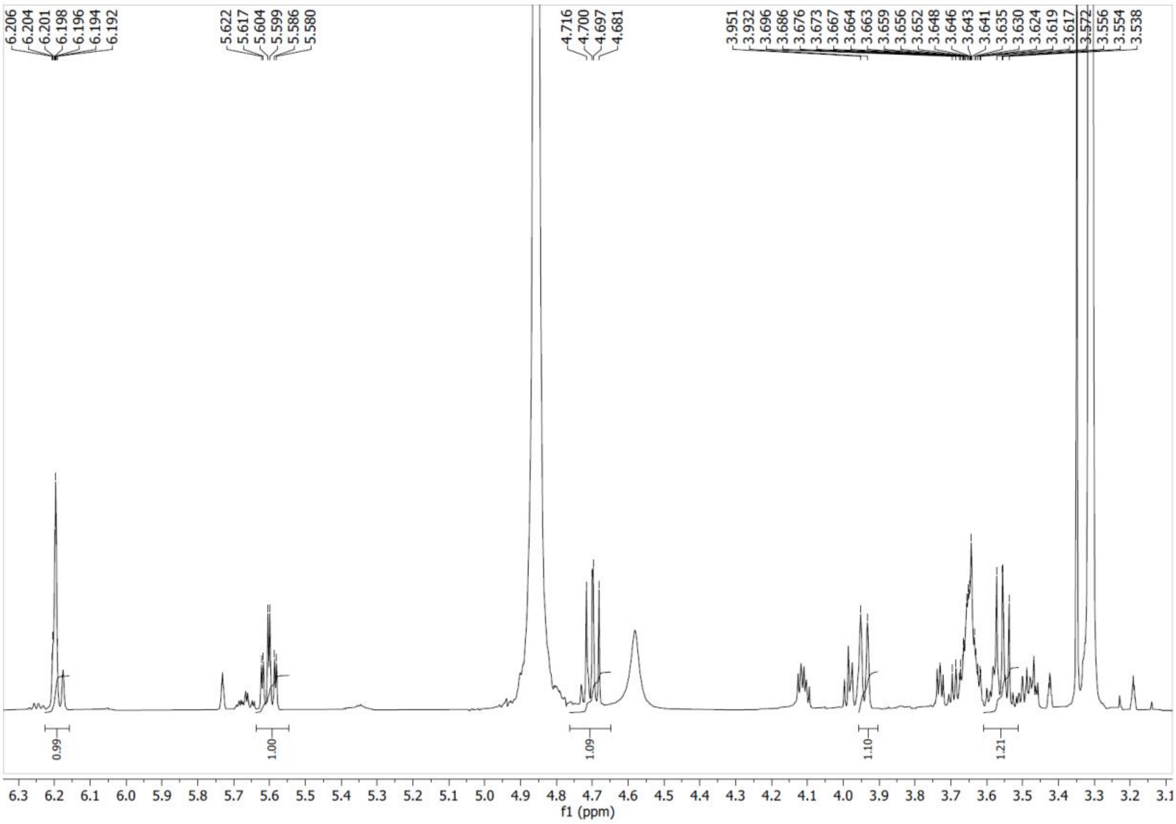

Fig. S10a. ^{1}H NMR spectrum of Ferupennin Q (**6**) in CD3OD at 600 MHz Zoom 1

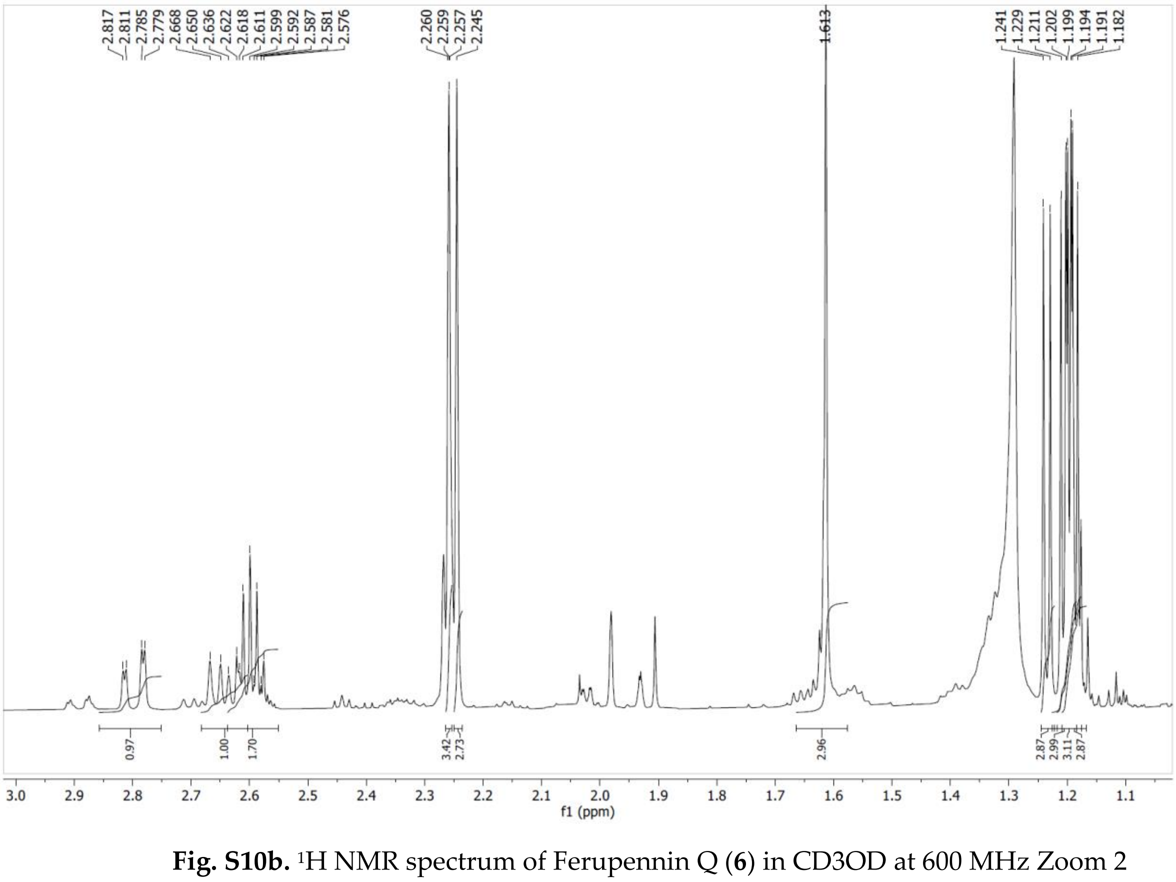

Fig. S10b. ^{1}H NMR spectrum of Ferupennin Q (**6**) in CD3OD at 600 MHz Zoom 2

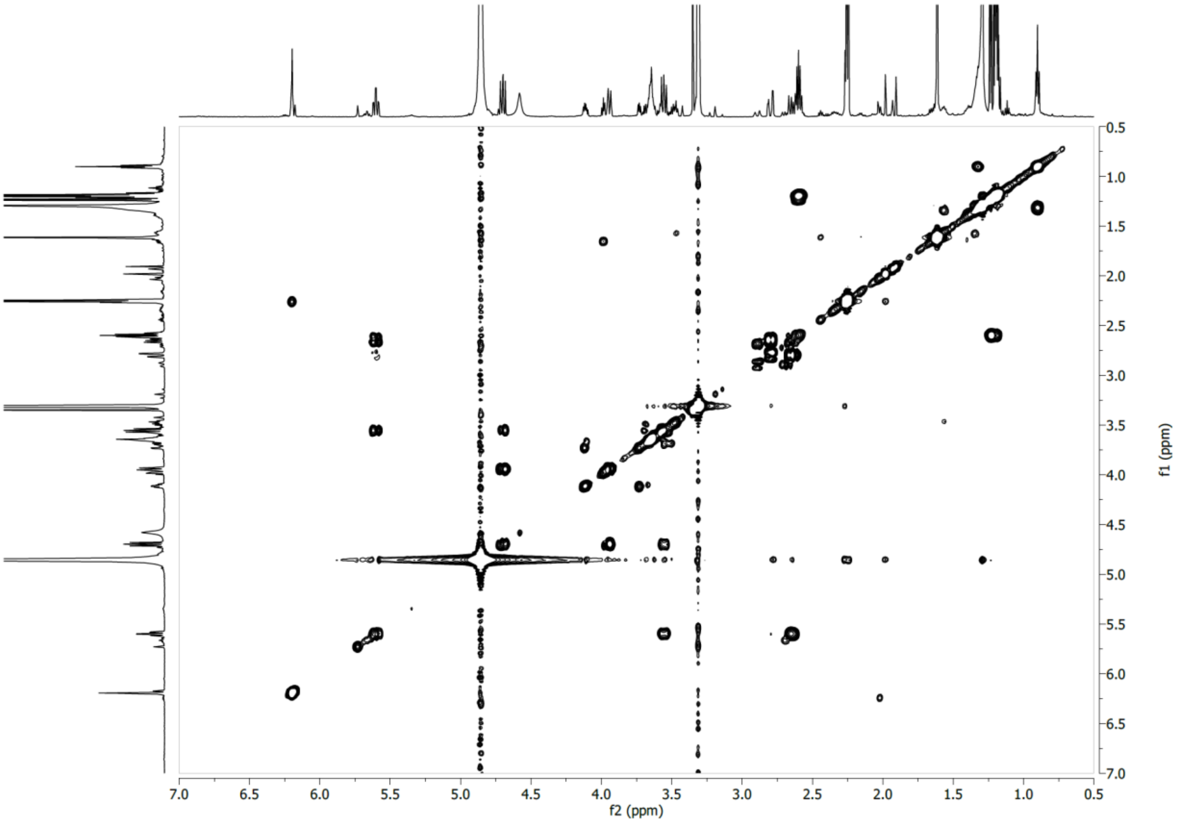

Fig. S11. COSY NMR spectrum of Ferupennin Q (**6**) in CD_3OD

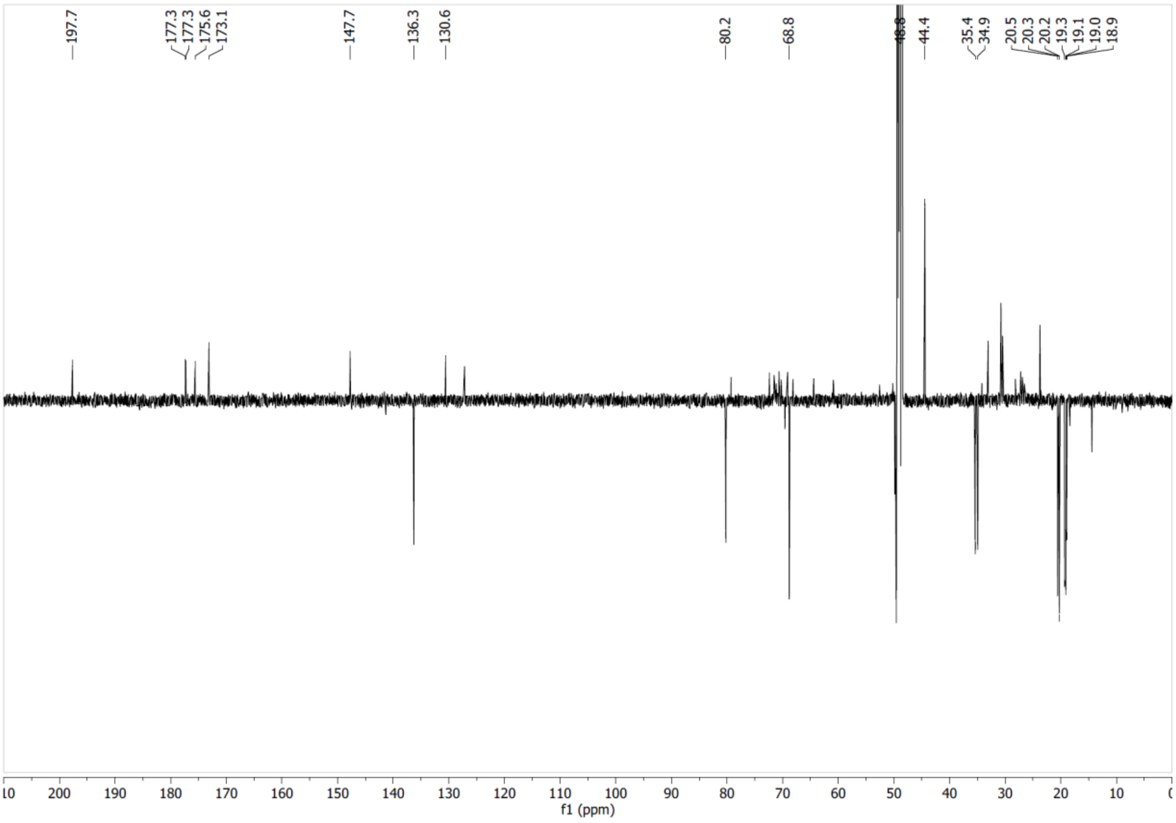

Fig. S12. ^{13}C-DEPTQ NMR spectrum of Ferupennin Q (**6**) in CD_3OD at 151 MHz

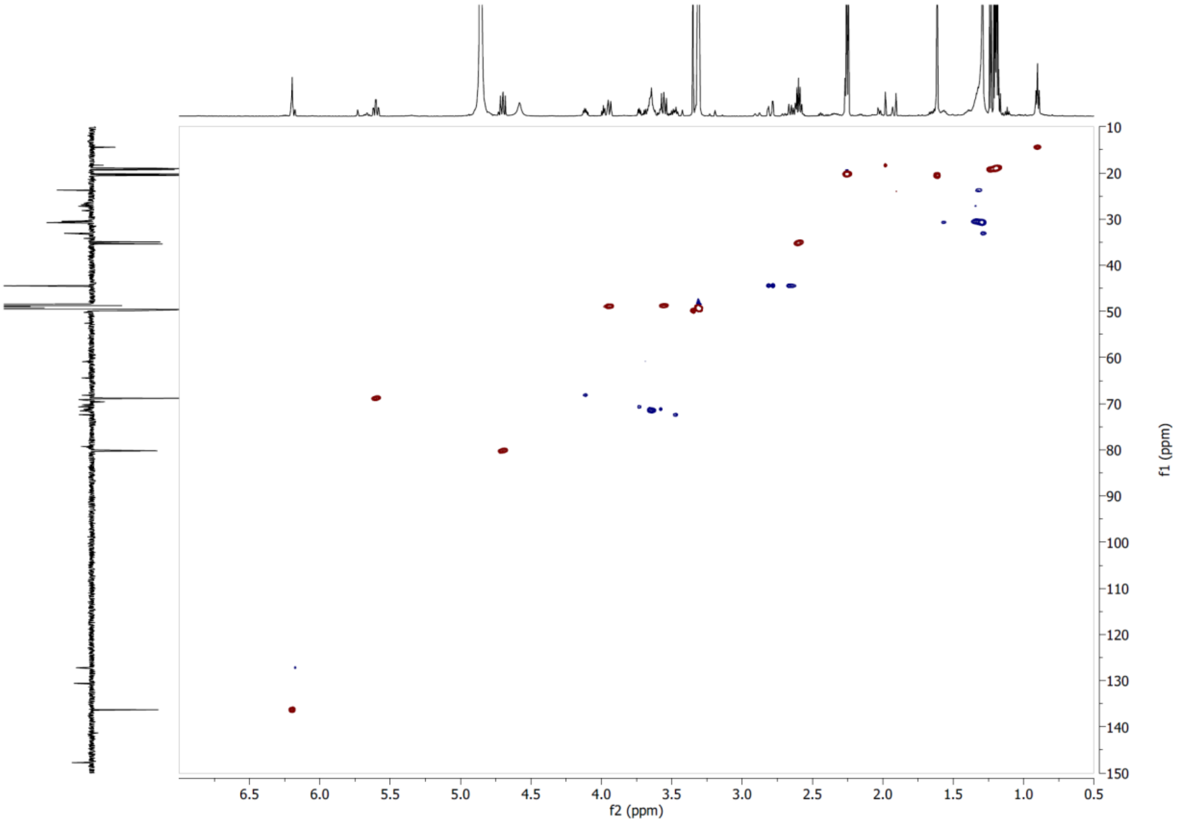

Fig. S13. Edited-HSQC NMR spectrum of Ferupennin Q (**6**) in CD_3OD

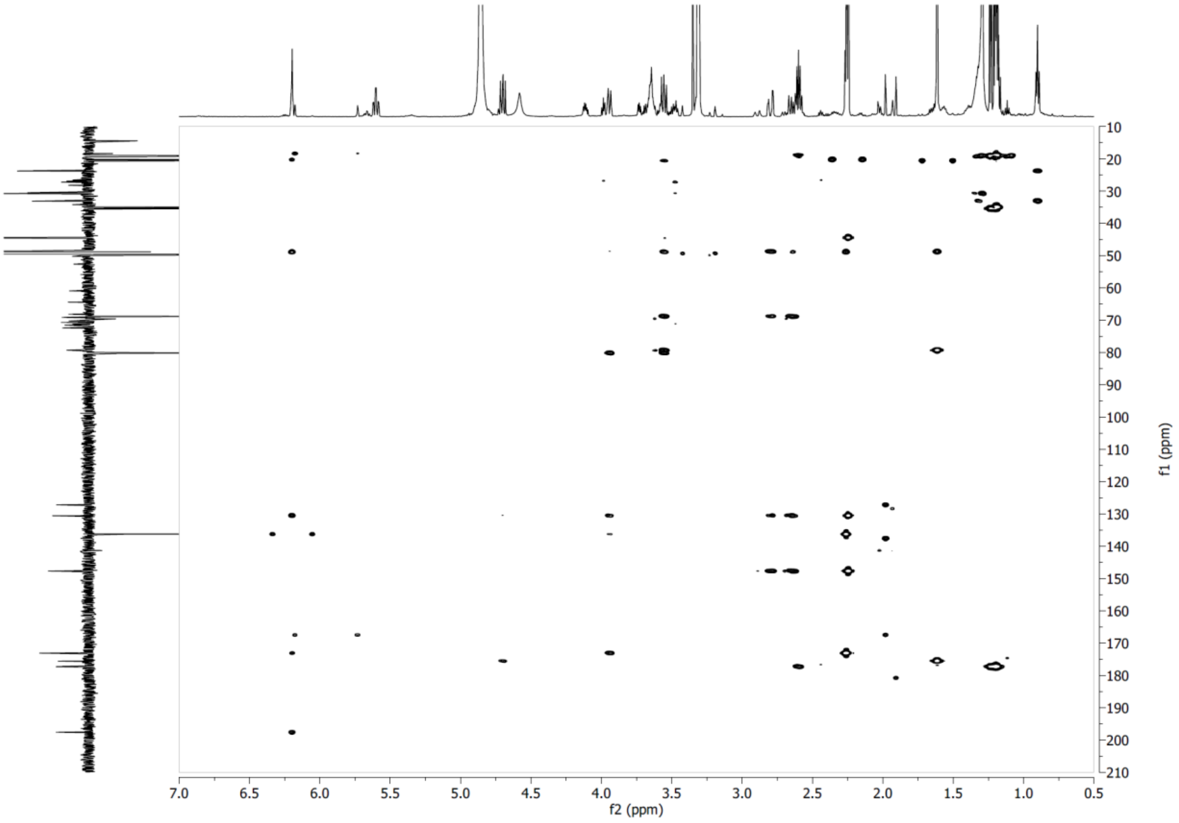

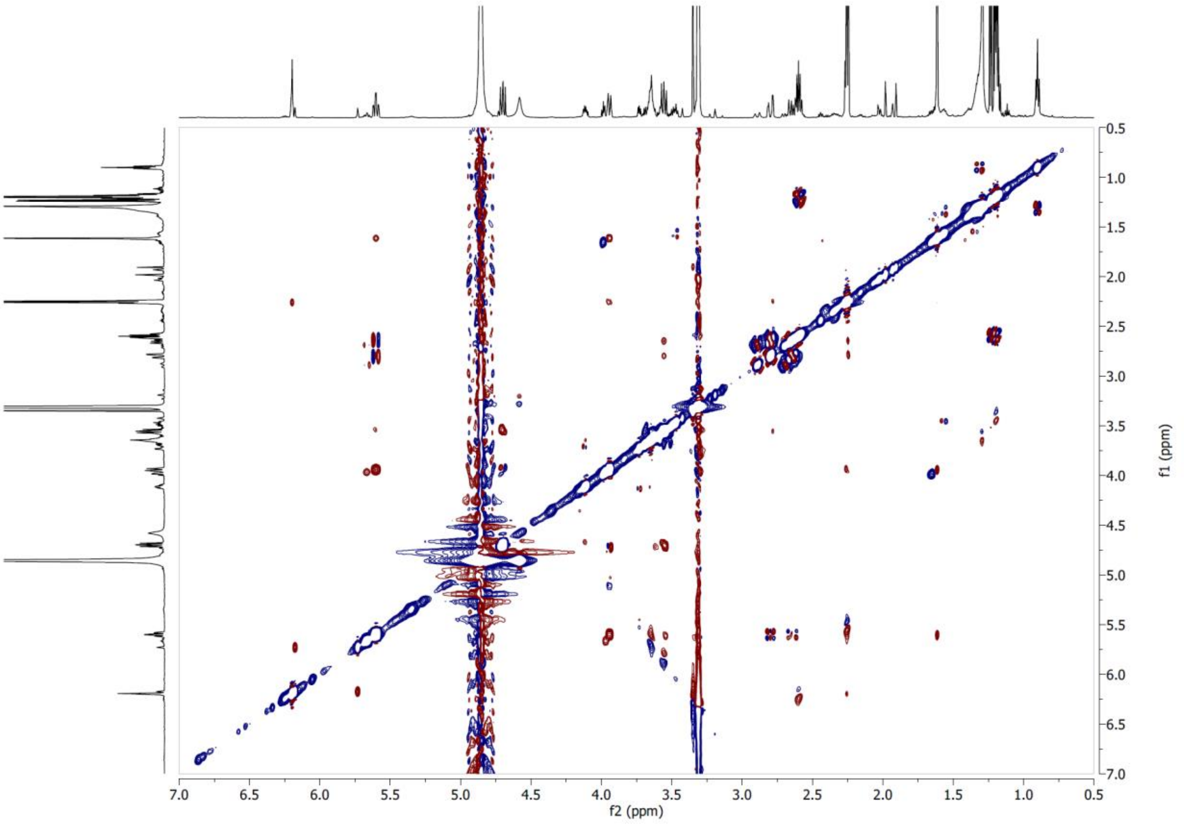

Fig. S14. ROESY NMR spectrum of Ferupennin Q (**6**) in CD_3OD

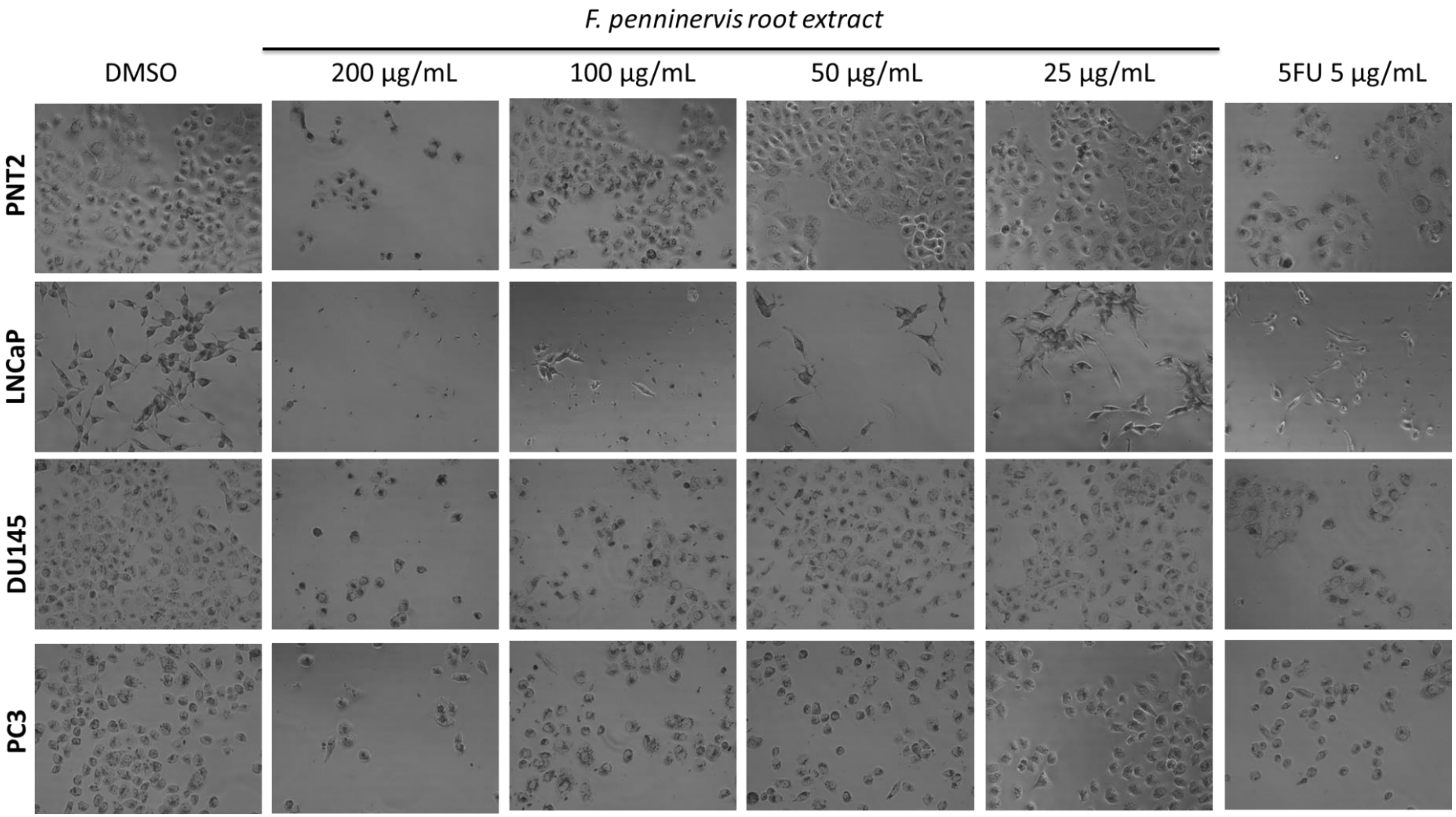

Fig. S15. Morphology of prostate epithelial cells PNT2 and prostate cancer cell lines LNCaP, DU145, PC3 cells grown for 48h with various concentrations of *F. penninervis* root extract, DMSO as a solvent control or 5-fluorouracil (5 µg/mL); neutral red staining, 10× magnification; pictures are representative for 3 experiments

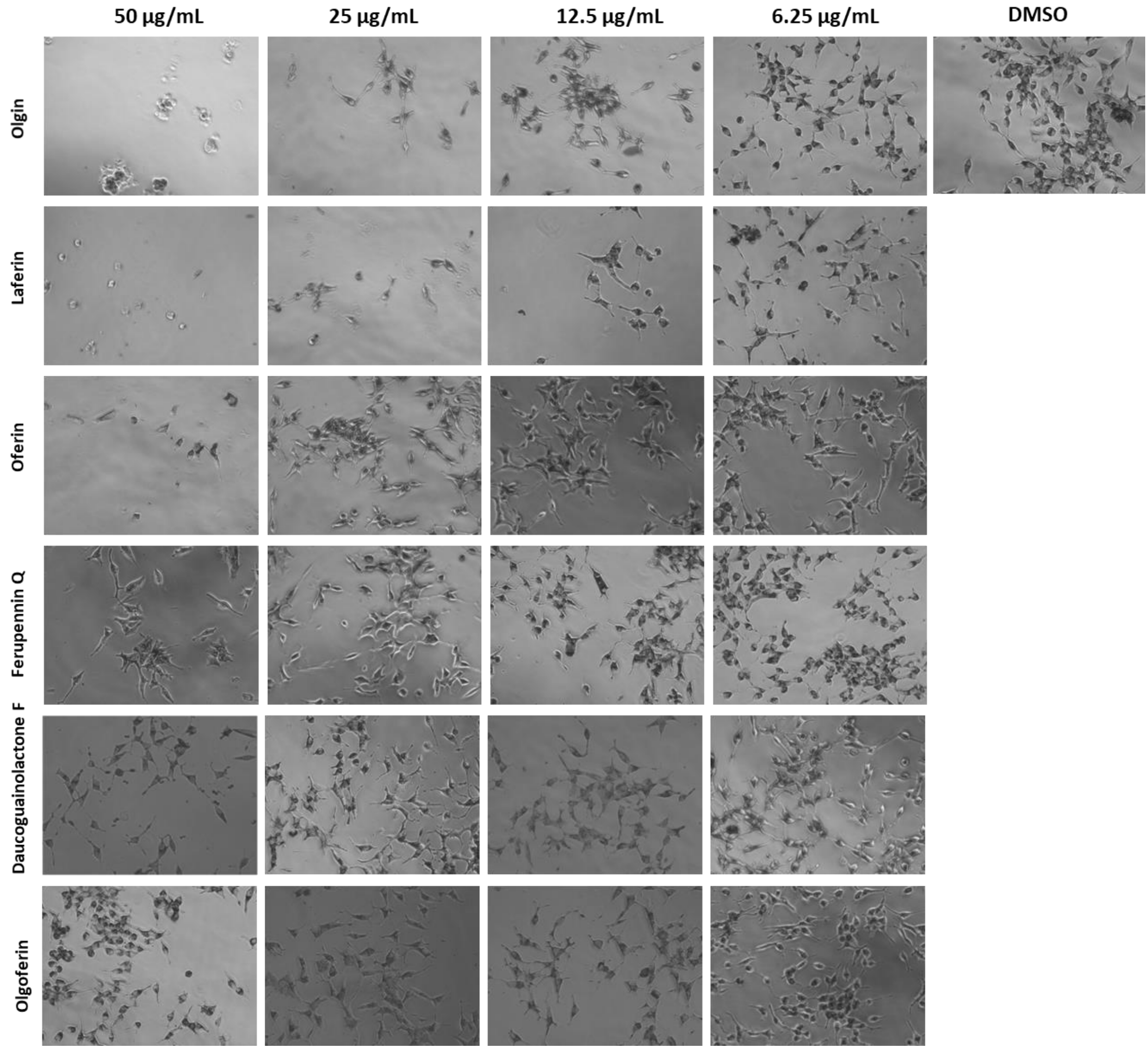

Fig. S16. Morphology of LNCaP prostate cancer cells grown for 48h with various concentrations of F. penninervis compounds or DMSO as a solvent control; neutral red staining, 10× magnification; pictures are representative for 3 experiments

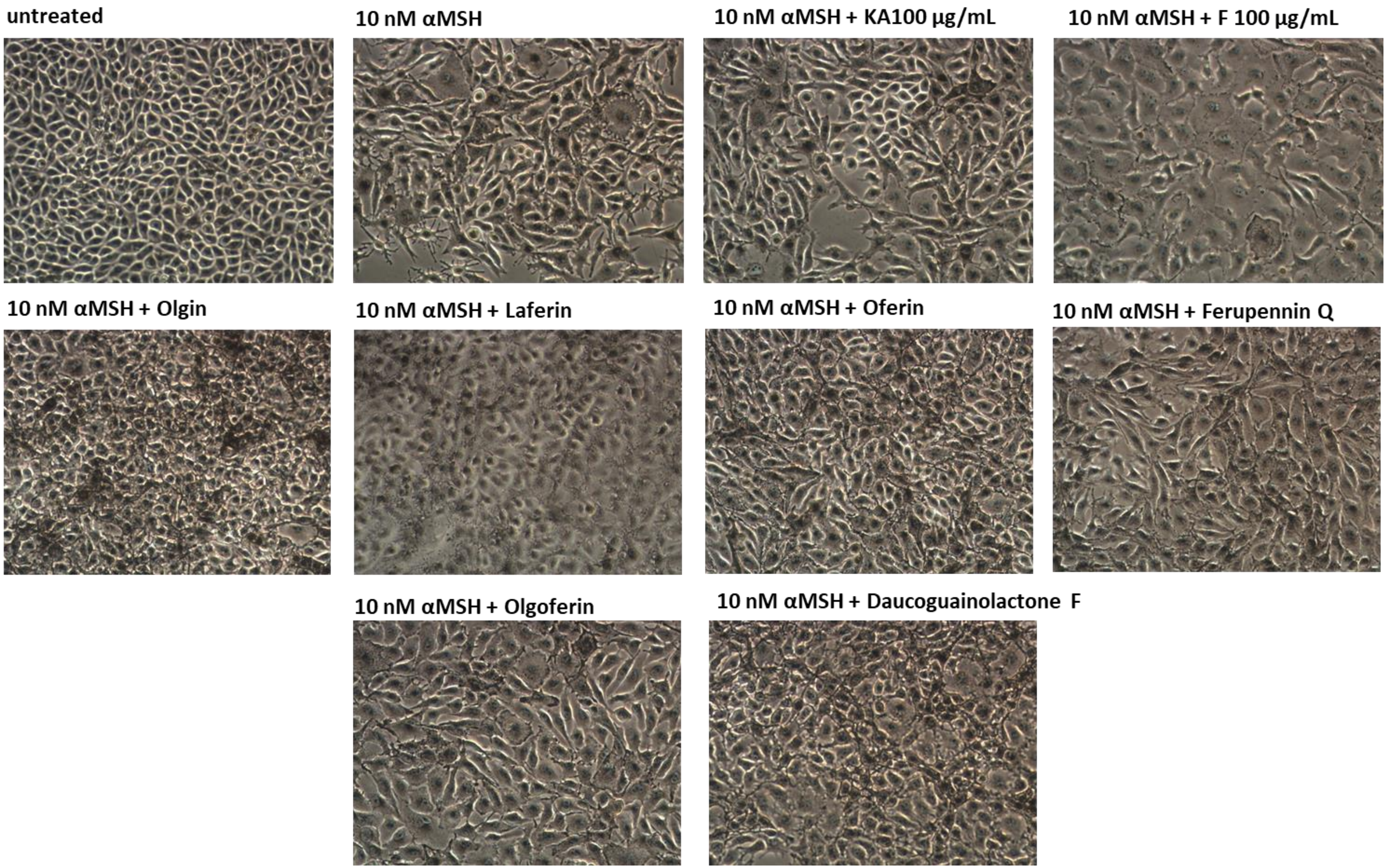

Fig. S17. Morphology of murine melanome B16F10 cells grown for 72 h with DMSO (untreated), αMSH (10 nM) and *F. penninervis* root extract (100 µg/mL), kojic acid (100 µg/mL) or isolated sesquiterpene lactones (10 µg/mL); neutral red staining, 10× magnification; pictures are representative for 3 experiments
